# Efficient CRISPR–Cas9 mediated multiplex genome editing in yeasts

**DOI:** 10.1186/s13068-018-1271-0

**Published:** 2018-10-10

**Authors:** Laiyou Wang, Aihua Deng, Yun Zhang, Shuwen Liu, Yong Liang, Hua Bai, Di Cui, Qidi Qiu, Xiuling Shang, Zhao Yang, Xiuping He, Tingyi Wen

**Affiliations:** 1 CAS Key Laboratory of Pathogenic Microbiology and Immunology, Institute of Microbiology, Chinese Academy of Sciences, Beijing, 100101 China; 2 University of Chinese Academy of Sciences, Beijing, 100049 China; 3 CAS Key Laboratory of Microbial Physiological and Metabolic Engineering, Institute of Microbiology, Chinese Academy of Sciences, Beijing, 100101 China; 4 Savaid Medical School, University of Chinese Academy of Sciences, Beijing, 100049 China

**Keywords:** CRISPR–Cas9-assisted multiplex genome editing, Markerless multi-locus integration, Markerless multi-copy integration, *Ogataea polymorpha*, *Saccharomyces cerevisiae*

## Abstract

**Background:**

The thermotolerant methylotrophic yeast *Ogataea polymorpha* has been regarded as an important organism for basic research and biotechnological applications. It is generally recognized as an efficient and safe cell factory in fermentative productions of chemicals, biofuels and other bio-products. However, it is difficult to genetically engineer for the deficiency of an efficient and versatile genome editing technology.

**Results:**

In this study, we developed a CRISPR–Cas9-assisted multiplex genome editing (CMGE) approach including multiplex genes knock-outs, multi-locus (ML) and multi-copy (MC) integration methods in yeasts. Based on CMGE, various genome modifications, including gene deletion, integration, and precise point mutation, were performed in *O. polymorpha*. Using the CMGE-ML integration method, three genes *TAL* from *Herpetosiphon aurantiacus*, *4CL* from *Arabidopsis thaliana* and *STS* from *Vitis vinifera* of resveratrol biosynthetic pathway were simultaneously integrated at three different loci, firstly achieving the biosynthesis of resveratrol in *O. polymorpha*. Using the CMGE-MC method, ∼ 10 copies of the fusion expression cassette *P*_*ScTEF1*_-*TAL*-*P*_*ScTPI1*_-*4CL*-*P*_*ScTEF2*_-*STS* were integrated into the genome. Resveratrol production was increased ~ 20 fold compared to the one copy integrant and reached 97.23 ± 4.84 mg/L. Moreover, the biosynthesis of human serum albumin and cadaverine were achieved in *O. polymorpha* using CMGE-MC to integrate genes *HSA* and *cadA*, respectively. In addition, the CMGE-MC method was successfully developed in *Saccharomyces cerevisiae*.

**Conclusions:**

An efficient and versatile multiplex genome editing method was developed in yeasts. The method would provide an efficient toolkit for genetic engineering and synthetic biology researches of *O. polymorpha* and other yeast species.

**Electronic supplementary material:**

The online version of this article (10.1186/s13068-018-1271-0) contains supplementary material, which is available to authorized users.

## Background

The thermotolerant methylotrophic yeast *Ogataea polymorpha* (*Hansenula polymorpha*), belonging to the fungal family of *Saccharomycetaceae*, subfamily *Saccharomycetoideae*, has been regarded as an attractive organism for fundamental and applied researches [1–5]. It has been an important organism for studies on methanol utilization, autophagy, peroxisome biogenesis and nitrate assimilation [6]. One attractive property of *O. polymorpha* is able to integrate up to 100 copies of target gene into the genome mediated by non-homologous end joining (NHEJ), which can be used to highly express heterologous genes and synthesize various biotechnology products [1, 4, 7, 8]. Furthermore, it can synthesize glycoproteins with human compatible oligosaccharides [9, 10]. In addition, it can grow at high temperatures up to 50 °C that would reduce the expensive cooling cost of industrial fermentation [9]. Due to these properties, many bio-products, such as ethanol, vaccines, uricase and glutathione, have been successfully synthesized in *O. polymorpha*. Currently, several prophylactic HBV vaccines have been marketed [1, 4, 7, 8].

A versatile multiplex genome editing method is essential in construction of yeast cell factories for various bio-products [11–16]. The Cre/*loxP*-based site-specific recombination, the *mazF*-based counter-selectable and the plasmid-based CRISPR–Cas9 systems have been developed for gene modifications in *O. polymorpha* [15–17]. The Cre/*loxP* system was reported to leave a scar (*lox72*) at the target locus after gene editing, which might interfere with the subsequent genetic manipulation [17]. To overcome the problem, Song et al. used the *mazF* as counter-selectable marker for markerless gene deletion [16]. Recently, the CRISPR–Cas9 system has been employed to establish genome editing method [18, 25], by which homologous recombination-mediated gene replacement and NHEJ-mediated gene disruption were performed in *O. polymorpha* [18]. In addition, the pUDP system (a plasmid-based CRISPR/Cas9 system that was applicable to several yeast species) co-expressed Cas9 and gRNA in one plasmid was also developed for gene disruption as reported by Juergens et al. [25]. Although available genome editing methods can adapt for gene deletion and disruption, multiplex gene editing is not possible in *O. polymorpha*. Therefore, an efficient and markerless genome editing system that can mediate multiplex genome engineering is urgently needed in *O. polymorpha*.

Recent advances in CRISPR–Cas9-assisted genome editing technologies provide an efficient approach to establish multiplex genome engineering [18–20]. In principal, a trans-activating crRNA (tracrRNA):crRNA duplex directs the Cas9 protein to bind to a target DNA sequence immediately followed by a protospacer adjacent motif (PAM). The Cas9 protein then makes a double-strand break (DSB) in three nucleotides upstream of the PAM site [18, 21–23]. The DSB can be repaired either by non-homologous end joining to result in insertions or deletions, or by homologous recombination to result in precise editing, such as gene deletion, point mutation and integration [5, 6, 17, 24].

Ribosomal DNA (rDNA) is a DNA sequence encoding ribosomal RNA. In yeasts, rDNA typically consists of high copy numbers of identical repeats that are clustered in a head-to-tail tandem array. Each rDNA repeat consists of two transcribed regions separately coding 35S precursor rRNA and 5S rRNA, and two non-transcribed regions NTS1 and NTS2 [25]. The number and the length of rDNA repeat vary among different species [26]. In *S. cerevisiae*, the rDNA locus consists of 150–200 tandem copies of 9.1-kb rDNA repeat [25]. In *O. polymorpha*, the rDNA contains about 50–60 copies of 8-kb unit [27]. Recently, rDNA repeats have served as target loci for multi-copy integrations in some yeast species [28–30].

In this study, we developed a CRISPR–Cas9-assisted multiplex genome editing method (designated as CMGE) for various gene modifications, especially for multiplex genes knock-outs, multi-locus (ML) and multi-copy (MC) integrations of target genes in *O. polymorpha.* Using CMGE, biosynthesis of resveratrol, cadaverine and human serum albumin (HSA) were achieved in *O. polymorpha*, suggesting the practicability and effectiveness of CMGE in genetic engineering of *O. polymorpha*. In addition, the CMGE-MC method was successfully developed in the model yeast *S. cerevisiae*.

## Results

### Scheme for CRISPR–Cas9-assisted genome editing in *O. polymorpha*

**Fig. 1:**
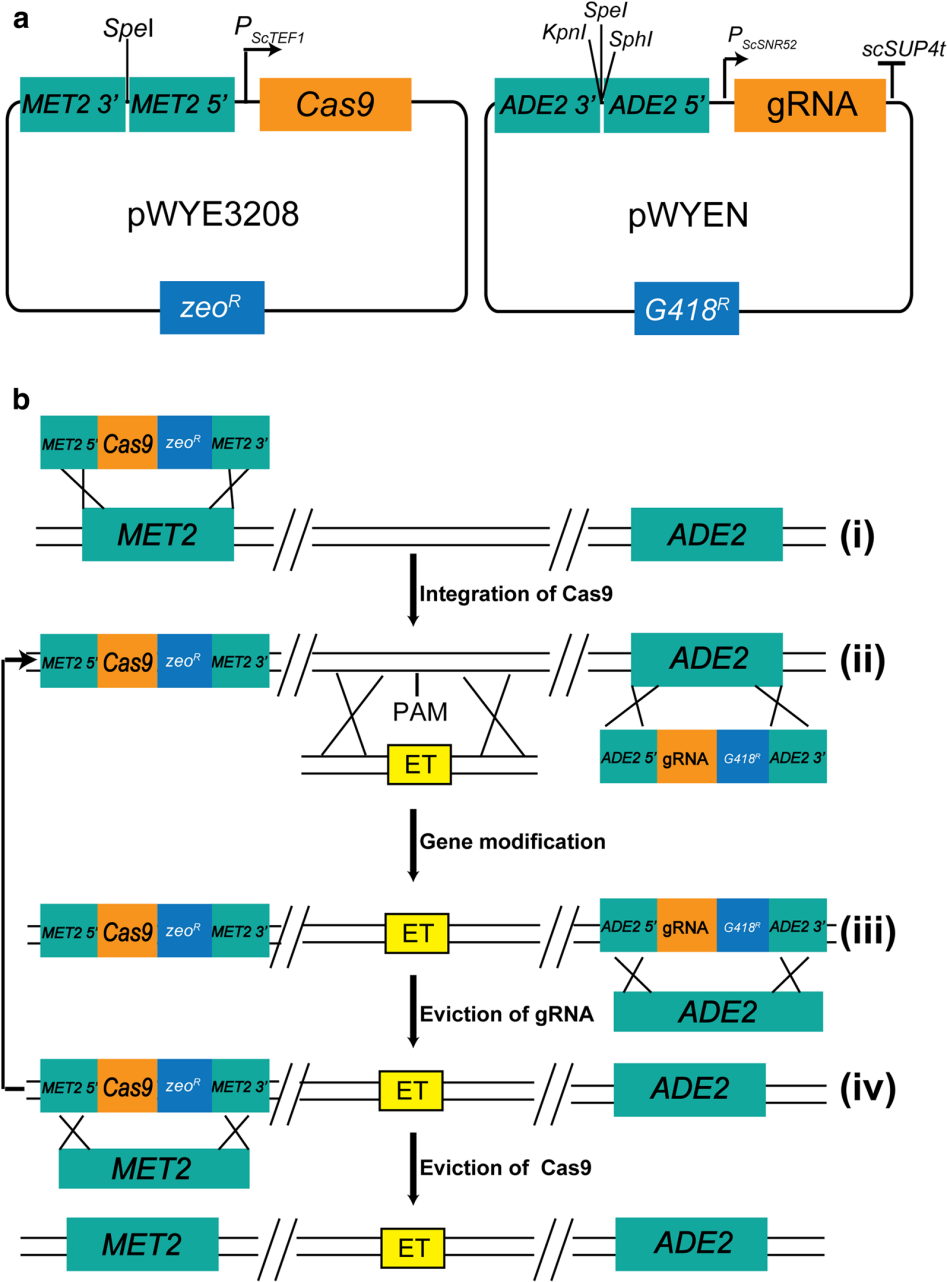
Schematic overview of the CRISPR–Cas9-assisted genome editing in *O. polymorpha.* **a** The Cas9 delivery vector pWYE3208. The expression of *Cas9* was driven by the promoter *P*_*ScTEF1*_. The *OpMET2* 5′ and *OpMET2* 3′ represent the ~ 1.5 kb of up- and downstream homologous arms of the gene *OpMET2*, respectively. *zeo*^*R*^ represents the zeocin resistance gene. The plasmid pWYEN is the gRNA delivery vector. The gRNA was driven by the promoter *P*_*ScSNR52*_ and ended with the terminator _*SC*_* SUP4t* . *OpADE2* 5′ and *OpADE2* 3′ represent the ~ 1.5 kb up- and downstream homologous arms of the gene *OpADE2*, respectively. *G418*^*R*^ represents the G418 resistance gene. **b** Schematic illustration of markerless genome editing. (i) Integration of *Cas9.* (ii) Gene modification. (iii) Eviction of the gRNA delivery vector and restoration of the *OpADE2* gene. (iv) Eviction of the *Cas9* delivery vector and restoration of the *OpMET2* gene. PAM is the abbreviation of protospacer adjacent motif. ET represents the editing template (s)

Due to unavailability of a stable episomal vector in *O. polymorpha*, the Cas9 and gRNA expression cassettes were integrated into the chromosome using recombinant plasmids pWYE3208 and pWYEN (a generic term of all gRNA delivery vector of which “N” represents the serial number. For example, “N” was 3208 in the plasmid pWYE3208), respectively (Fig. 1a). The plasmid pWYE3208 harboring the *P*_*ScTEF1*_-controlled *cas9* gene and the up- and downstream homologous arms (UHA and DHA) of the *OpMET2* gene was linearized with *Spe*I and then transformed into *O. polymorpha*. Through a double-crossover homologous recombination, the *OpMET2* gene was replaced by the linearized pWYE3208 (Fig. 1b). The desired mutant OP009 (Zeo^R^, OP001Δ*OpMET2*::*P*_*ScTEF1*_-*Cas9*) was verified on YPD plate containing zeocin and further confirmed by PCR.

The gRNA expression cassette was composed of the promoter *P*_*scSNR52*_, N_20_, trans::crRNA duplex and the terminator _*SC*_*SUP4t*. To conveniently screen transformants harboring gRNA expression cassette, *OpADE2* was selected as the integration site of gRNA expression cassette due to the formation of red colonies by accumulating the oxidized 5-amino imidazole ribonucleotide.

Three unique restriction sites (*Spe*I/*Kpn*I/*Sph*I) between the UHA and DHA of *OpADE2* gene were used to linearize plasmid (Fig. 1a). After co-transformed into the strain OP009 with the editing template, the linearized gRNA delivery vector transcribed gRNA that can guide the Cas9 protein to recognize the PAM site. The Cas9 protein cleaved double DNA strands to generate the DSB, which was subsequently repaired by the editing template via homologous recombination. Meanwhile, the gRNA delivery vector was integrated at the gene *OpADE2* locus by homologous recombination (Fig. 1b).

The resulting transformants were screened on YPD plates containing G418. The desired mutants were obtained by two-step procedures: (i) red colonies were selected to identify the integration of gRNA expression cassette at the *OpADE2* locus by PCR; (ii) The positive transformants were randomly selected from red colonies to identify the desired mutation at the target site of gRNA.

Because the integration of gRNA delivery vector was mediated by endogenous homologous recombination system (HRS), the fragment *OpADE2*UHA (~ 1.5 kb)-*G418*^*R*^ expression cassette-*OpADE2*DHA (~ 1.5 kb) was transformed into OP009 to detect the editing efficiency mediated by the endogenous HRS. As shown in Additional file 1: Figure S1, the editing efficiency of 29.49 ± 4.44% was obtained. Given that 200–300 colonies could be obtained by transformation of 1 µg DNA, appropriately 60–90 red colonies would be obtained in the first step of screening.

After the desired mutation was verified, the gRNA delivery vector containing the marker gene *G418*^*R*^ was evicted to restore the native *OpADE2* locus by transforming with the *OpADE2* editing template (Fig. 1b). The resulting transformants were selected on the SC-ADE plate and further confirmed by PCR analysis and DNA sequencing. The mutant could be used for the next round of genome editing.

When all gene manipulations were accomplished, the Cas9 expression vector containing the marker gene *zeo*^*R*^ was evicted to restore the native *OpMET2* locus by transforming with the *OpMET2* editing template (Fig. 1b). The resulting transformants were verified on SC-MET plate and further confirmed by PCR and DNA sequencing.

### Gene deletion

**Fig. 2:**
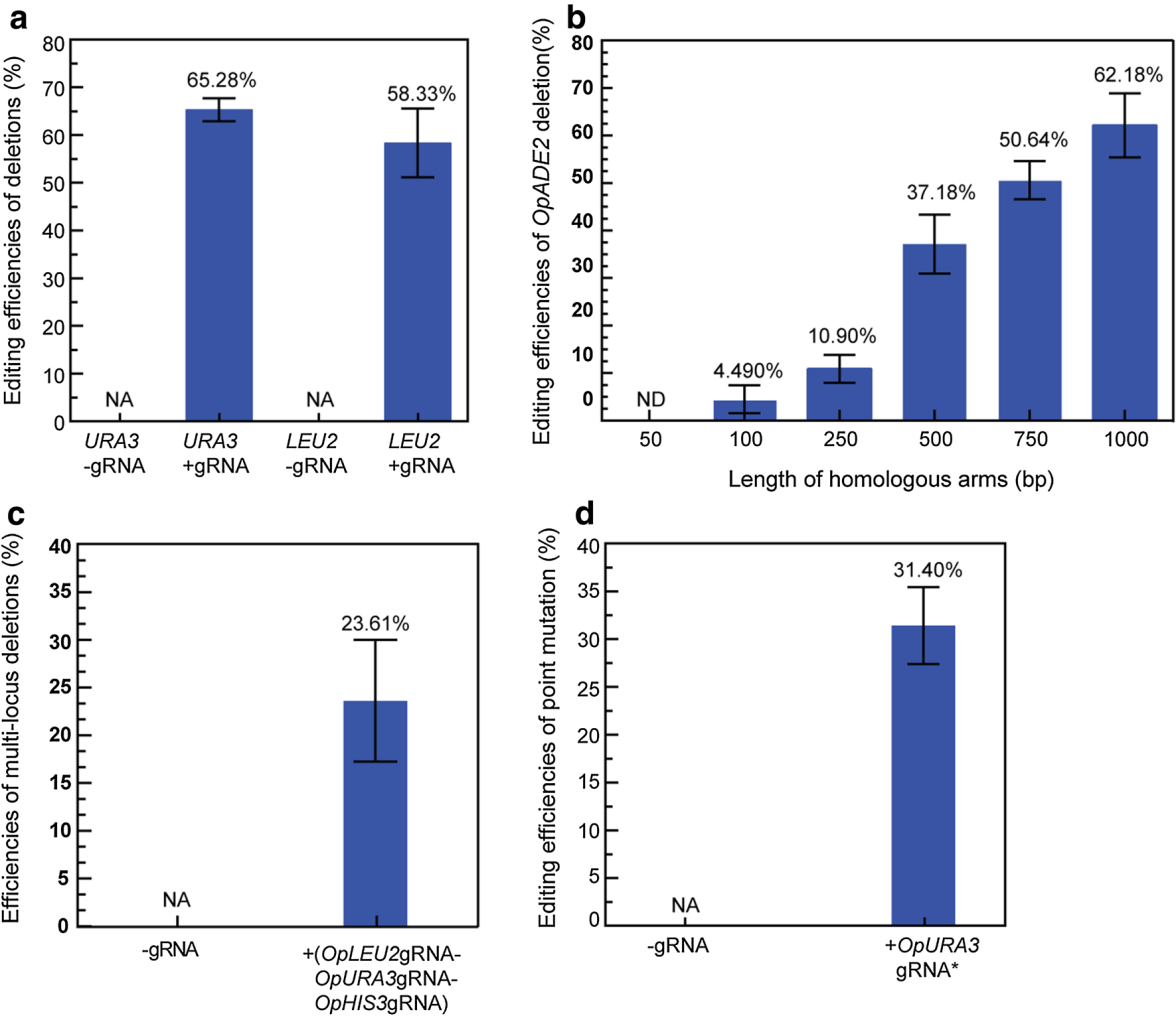
Gene deletion and point mutation in *O. polymorpha*. **a** Editing efficiencies for deletions of genes *OpURA3* and *OpLEU2*. **b** Editing efficiencies of *OpADE2* deletion using 50- to 1000-bp length of homologous arms. **c** Editing efficiencies of multi-locus deletions of three genes *OpLEU2, OpURA3 and OpHIS3*. **d** The editing efficiency of point mutation in the gene *OpURA3*. NA: no correct mutant was obtained. Error bars indicate standard deviations of three biological repeats (n = 3)

To validate the effectiveness of CRISPR–Cas9 mediated genome editing system in markerless gene deletion, two genes *OpLEU2* encoding the 3-isopropylmalate dehydrogenase and *OpURA3* encoding the orotidine-5′-phosphate decarboxylase were separately selected as targets. Following the described procedure in Fig. 1b, eight colonies for each mutant were randomly selected from red-color colonies to detect editing efficiencies. Editing efficiencies for deletion of *OpLEU2* and *OpURA3* were 58.33 ± 7.22% and 65.28 ± 2.41%, respectively (Fig. 2a). In addition, homologous recombination frequencies without CRISPR/Cas9-induced DSB were too low to obtain the desired mutant in the control experiment by co-transformating with the vector without gRNA and the editing template into the OP009 strain (Fig. 2a; Additional file 1: Figure S2A, B). Auxotrophic phenotype analysis showed that *OpURA3* or *OpLEU2*-deficient strains were unable to grow on SC-URA or SC-LEU medium, further confirming inactivation of the *OpURA3* or *OpLEU2* in *O. polymorpha* (Additional file 1: Figure S4A, B). Finally, the linearized gRNA delivery vector and the Cas9 protein expression cassette were successively evicted from the chromosome as described procedure in Fig. 1b (Additional file 1: Figure S3A, B).

In addition, the effect of homolog size on editing efficiency was tested. The gene *OpADE2* was selected as the target gene for deletion and the gene *OpURA3* was selected as the target gene for integration of the gRNA expression cassette (details were described in methods in Additional file 1). Various editing templates with different size of homologous arms (50, 100, 250, 500, 750 and 1000 bp) were used. As shown in Fig. 2b, the editing efficiency increased with the length of homologous arm, which was consistent with the effects of homologous arm sizes on gene editing efficiency in the previous report [31]. No corrected mutant was obtained when the HA was 50 bp, while the editing efficiency reached 62.18 ± 6.17% when HA was 1000 bp (Additional file 1^1^ Figure S5). Furthermore, the editing efficiency decreased drastically when the homologous arm size was less than 500 bp. Therefore, the homologous arm size of editing template should be no less than 500 bp in the design process.

To achieve simultaneous multiple knock-outs in *O. polymorpha,* three genes *OpURA3*, *OpHIS3* and *OpLEU2* were selected as the target loci. The UHA and DHA (~ 1 kb) fragments of each target gene were joined by Splicing Overlapping Extension PCR as editing template. Three different editing templates for deletions of *OpURA3*, *OpHIS3* and *OpLEU2* were co-transformed into the strain OP009 with the linearized vector pWYE3215 harboring three gRNAs expression cassettes to separately target three genes. The vector without gRNA was used as a control. The desired mutant was obtained with the editing efficiency of 23.61 ± 6.36% by PCR analysis (Fig. 2c and Additional file 1: Figure S6). Auxotrophic phenotype analysis showed that the strain OP045 (OP001Δ*OpLEU2*Δ*OpHIS3*Δ*OpURA3*) was unable to grow on SC-LEU, SC-HIS3 and SC-URA plates, further confirming simultaneously inactivation of the *OpLEU2, OpHIS3* and *OpURA3* in *O. polymorpha* (Additional file 1: Figure S7).

### Precise point mutation

To test the applicability of the genome editing system in precise nucleotide substitution, G73T mutation was introduced into the *OpURA3* gene. The mutation site G73 is designed in the third nucleotide of the PAM sequence TGG to eliminate the PAM sequence upon successful mutation, thus preventing the Cas9 nuclease from breaking the mutated sequence. The substitution of nucleotide G by T resulted in the introduction of a stop codon into the *OpURA3* gene at mutation site, which would make mutants fail to grow on SC-URA plate.

The editing template was a DNA fragment (~ 2 kb) containing the mutant *OpURA3* gene (G73T), and its up- and downstream homologous arms. The editing template and linearized plasmid pWYE3211 (*OpADE2*upHA-*P*_*ScSNR52*_-*OpURA3*gRNA^*^-*OpADE2*downHA) used to transcribe gRNA were co-transformed into the strain OP009 (OP001Δ*OpMET2*::*P*_*ScTEF1*_-*Cas9*). The resulting transformants were screened on YPD plates containing G418. Desired mutants were selected from transformants by uracil auxotrophic phenotype on SC-URA plates (Additional file 1: Figure S8).

Desired mutants were generated with the editing efficiency of 31.40 ± 4.02% (Fig. 2d). The mutation site was confirmed by the nucleotide sequence of the gene *OpURA3* and the uracil auxotrophic phenotype (Additional file 1: Figure S9). To detect whether there were off-target mutations, the web-based computer program CAS-OFFinder was used to predict potential off-target sites (Additional file 1: Table S3). All potential off-target sites in the mutant OP040 (OP001*OpURA3*^G73T^) were sequenced and the off-target mutation was not detected (Additional file 1: Figure S10). The results suggested that the CRISPR–Cas9-assisted system developed in this study could be used to precisely introduce a nucleotide mutation in the genome.

### Multi-locus gene integration

**Fig. 3:**
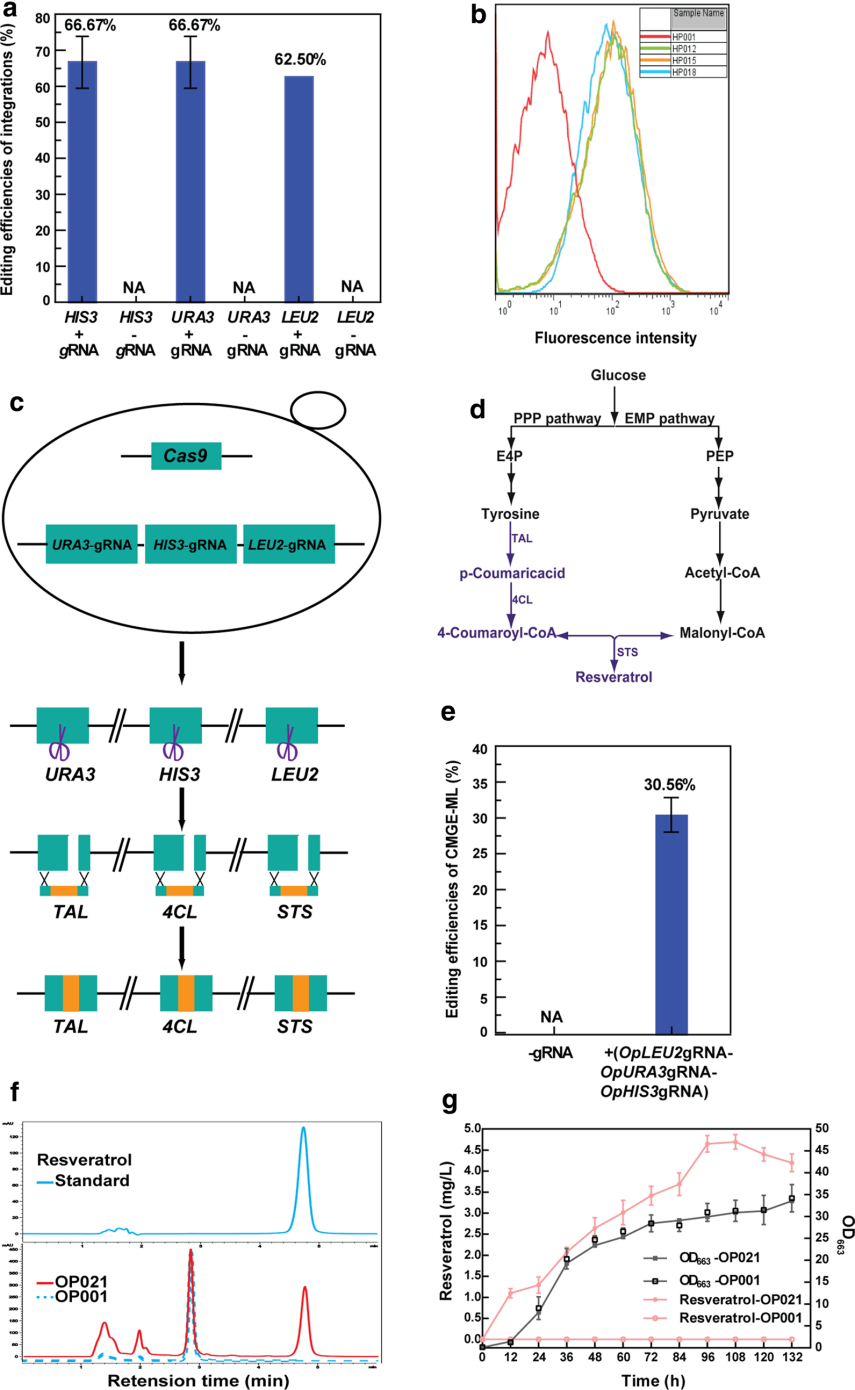
Multi-locus integration. **a** Editing efficiencies of *gfpmut3a* at three different loci. **b** Flow cytometry analysis of the expression of GFP in strains OP012 (OP001Δ*OpLEU2*::*gfpmut3a*), OP015 (OP001Δ*OpURA3*::*gfpmut3a*) and OP018 (OP001Δ*OpHIS3*::*gfpmut3a*). c The sketch map of simultaneous integrations of *TAL*, *4CL* and *STS* genes at *OpURA3*, *OpHIS3* and *OpLEU2* loci, respectively. **d** Biosynthetic pathway of resveratrol by integrating *TAL*, *4CL* and *STS* genes (in blue). **e** Editing efficiencies of multi-locus integrations with and without the expression of targeting gRNAs. **f** HPLC analysis of resveratrol. **g** Cell growth and resveratrol productions of the mutant strain OP021 (OP001Δ*OpHIS3*::*4CL* Δ*OpURA3*::*TAL* Δ*OpLEU2*::*STS*) and the wild-type strain

Three genes *OpLEU2*, *OpURA3* and *OpHIS3* encoding the imidazoleglycerol-phosphate dehydratase were selected as target genes to further evaluate the feasibility of the CRISPR–Cas9 mediated genome editing system for gene integration. The *gfpmut3a* expression cassette flanked by UHA and DHA of the target gene was used as the editing template for single site. The editing efficiencies for integrations at *OpHIS3* and *OpURA3* loci were both 66.70 ± 7.22%, while that at *OpLEU2* locus was 62.50% (Fig. 3a; Additional file 1: Figure S11A–C).

To detect editing efficiencies mediated by endogenous HRS at three loci, the fragment(s) *OpLEU2* (or *OpURA3/HIS3*)UHA (~ 1 kb)-*G418*^*R*^ expression cassette-DHA (~ 1 kb) was transformed into OP009. As shown in Additional file 1: Figure S12A–C, editing efficiencies mediated by endogenous HRS were only 16.03 ± 1.11% (*OpLEU2*), 17.95 ± 4.84% (*OpURA3*) and 16.67 ± 1.11% (*OpHIS3*), respectively. Therefore, CRISPR/Cas9-induced DSB significantly enhances the editing efficiency in *O. polymorpha* (Additional file 1: Figure S-15). Moreover, the expression of the *gfpmut3a* gene in the mutant was further confirmed by flow cytometry analysis (Fig. 3b).

For testing simultaneous integration of multiple genes into the genome, the non-native synthetic pathway of resveratrol was introduced in *O. polymorpha* (Fig. 3d). Three different editing templates for integration of *TAL*, *4CL* and *STS* expression cassettes at *OpURA3*, *OpHIS3* and *OpLEU2* loci were co-transformed into the strain OP009 with the linearized vector pWYE3215 harboring three gRNAs expression cassettes to separately target three loci (Fig. 3c, d). The vector without gRNA was used as a control. The mutant integrating three genes was obtained with the editing efficiency of 30.56 ± 2.40% by phenotypic screening and PCR analysis (Fig. 3e and Additional file 1: Figure S14). The maximum resveratrol production was 4.69 ± 0.17 mg/L in the shake-flask cultivation of OP021 strain (Fig. 3f, g), achieving the first biosynthesis of resveratrol in *O. polymorpha.* Therefore, the CMGE-ML was effective in simultaneous integration of multiple genes into the genome of *O. polymorpha*.

### Multi-copy integration into rDNA cluster

**Fig. 4:**
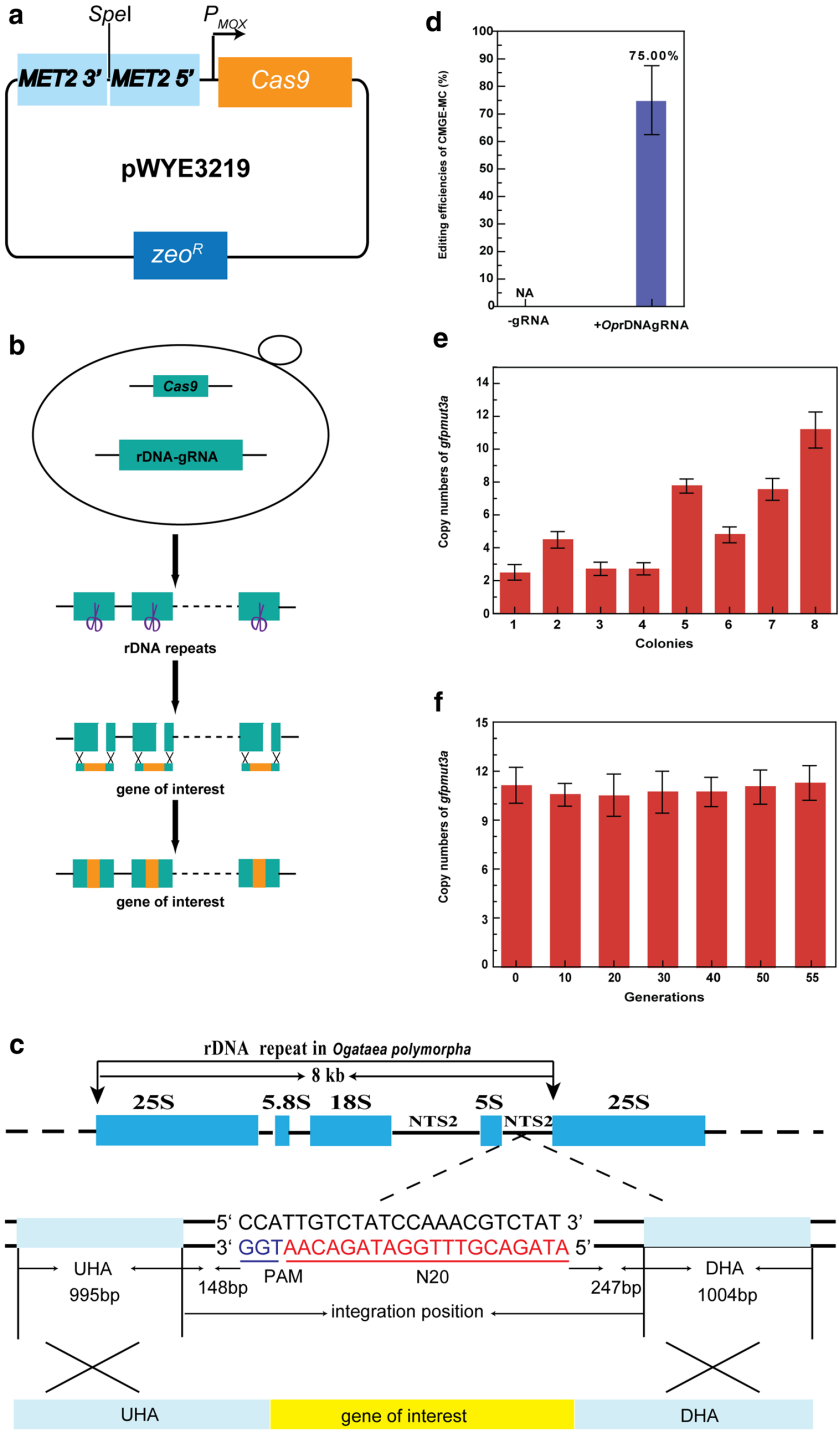
Multi-copy integration of *gfpmut3a* at rDNA repeats of *O. polymorpha*. **a** The Cas9 delivery vector pWYE3219. The expression of *Cas9* was driven by the inducible promoter *P*_*OpMOX*_. **b** A schematic illustration of integration of multi-copy integration at rDNA repeats. **c** Precise integration site of exogenous gene at rDNA cluster in *O. polymorpha.* **d** Integration efficiencies of *gfpmut3a* at rDNA repeats with and without the expression of targeting gRNA. Error bars indicate standard deviations of three biological repeats. **e** Copy numbers of *gfpmut3a* in eight randomly selected colonies. **f** Stability of multi-copy integration of *gfpmut3a* at rDNA repeats in the mutant OP025 (OP001rDNA::*gfpmut3a*)

To conduct multi-copy integration, the expression of *Cas9* was controlled by the inducible promoter *P*_Mox_ and the rDNA cluster consisting of 50–60 repeats were selected as integration sites (Fig. 4a). After inducible expression of *Cas9*, the linearized vector pWYE3220 harboring the gRNA expression cassette targeting rDNA repeats and editing template containing *gfpmut3a* expression cassette flanked by ~ 1 kb up- and downstream homologous arms to the target site were co-transformed into cells. DSBs at some of rDNA repeats were generated by the slicing of Cas9 and repaired by homologous recombination of donor DNA (Fig. 4b, c). Then, the expression of *Cas9* was suppressed by glucose in the resuscitation medium to avoid the slicing of Cas9 at the left rDNA repeats. As a result, mutants harboring *gfpmut3a* expression cassette at rDNA repeats were obtained with the editing efficiency of 75.00 ± 12.5% (Fig. 4d and Additional file 1: Figure S15).

Eight colonies were randomly selected to determine the copy numbers of *gfpmut3a* gene in the genome. The copy numbers arranged from 2.42 ± 0.47 (colony 1) to 11.15 ± 1.10 (colony 8) (Fig. 4e). To detect the expression of *gfpmut3a*, the GFP intensities of mutants harboring different copies of *gfpmut3a* were measured. As a result, all mutants exhibited green fluorescence and the fluorescence intensity showed an increased tendency with the increase of gene copy number (Fig. 4e and Additional file 1: Figure S16).

To evaluate the stability of multi-copy gene in the chromosome, the OP025 strain harboring 11.15 ± 1.10 copies of *gfpmut3a* was cultured in YPD medium without any selective pressure. As shown in Additional file 1: Figure S17, copy numbers of the *gfpmut3a* were constant over 96-h cultivation. Furthermore, to evaluate the stability of multiplex editing in a long-term lab evolution, the strain OP025 was cultured in YPD medium without any selective pressure for 18.5 days (55 generations). As shown in Fig. 4f, copy numbers of the *gfpmut3a* gene were constant after long-term propagation. The result suggested that rDNA repeats provided a useful and stable target site for integration of multiple heterologous genes.

**Fig. 5:**
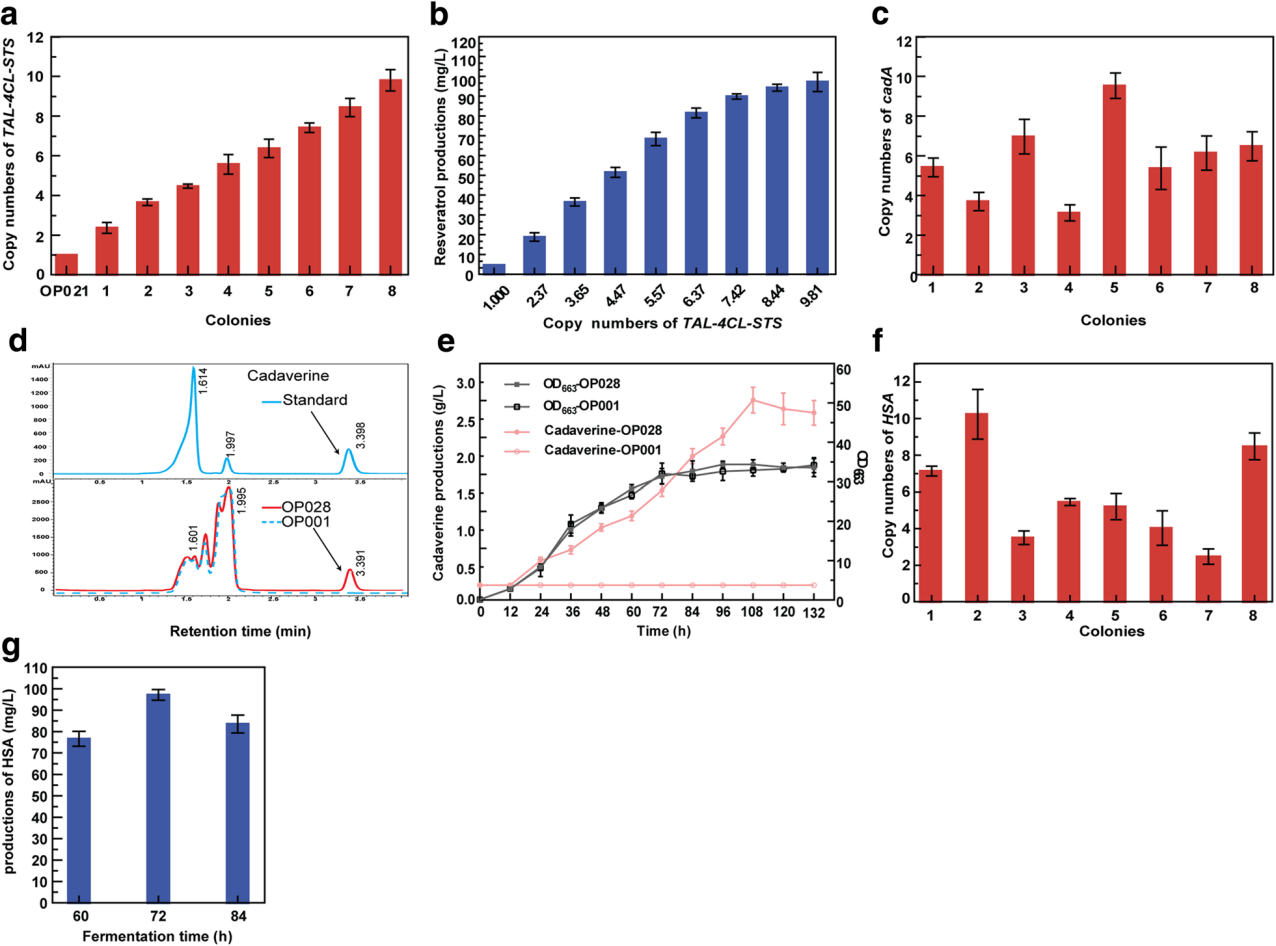
The application of multi-copy integration method in *O. polymorpha*. **a** Copy numbers of the fusion expression cassette *P*_*ScTEF1*_-*TAL*-*P*_*ScTPI1*_-*4CL*-*P*_*ScTEF2*_-*STS* integrated at rDNA cluster. **b** Resveratrol productions of colonies harboring different copy numbers of the fusion expression cassette *P*_*ScTEF1*_-*TAL*-*P*_*ScTPI1*_-*4CL*-*P*_*ScTEF2*_-*STS*. **c** Copy numbers of *cadA* integrated at rDNA cluster. **d** HPLC analysis of cadaverine in *O. polymorpha*. **e** Cell growth and cadaverine productions of the wild-type strain OP001 and the mutant OP028 (OP001rDNA::*cadA*). **f** Copy numbers of gene *HSA* integrated at rDNA cluster in eight randomly selected colonies. **g** HSA productions of the mutant strain OP031(OP001rDNA::*HSA*) by shake flask fermentation at different fermentation time

To demonstrate the utility and generality of CMGE-MC, three genes of *TAL*, *4CL* and *STS* were constructed into one fusion expression cassette (*P*_*ScTEF1*_-*TAL*-*P*_*ScTPI1*_-*4CL*-*P*_*ScTEF2*_-*STS*), which were then integrated into rDNA repeats. Integrants harboring different copy numbers of the expression cassette were obtained. To analyze the correlation between resveratrol production and copy number of target genes, integrants harboring one to ~ 10 copies were selected to culture in shake flask (Fig. 5a). As shown in Fig. 5b, the resveratrol production increased with the increase of gene copy numbers. The OP043 strain with 9.81 ± 0.55 copies yielded the highest production of 97.23 ± 4.84 mg/L resveratrol, which increased by 20.73-fold compared to that of the single-copy integrant. The result suggested that the more copies of expression cassette were integrated into the genome, the higher level of resveratrol production was achieved.  In addition, the resveratrol production increased slowly when the integrated gene reached 7-10 copies , indicating that the resveratrol production in *O. polymorpha* might be limited by other factors, such as the supply of precursor malonyl-CoA [32].

To further assess the broad applicability of CMGE-MC, the *cadA* gene from *E. coli* and the human serum albumin gene *HSA* were separately integrated into the multiple rDNA repeats. Mutants harboring different copy numbers (from 3.12 ± 0.41 to 9.54 ± 0.64) of *cadA* gene were obtained (Fig. 5c). After shake-flask cultivation of the stain OP028 harboring 9.54 ± 0.64 copies in the presence of lysine for 108 h, 2.51 ± 0.18 g/L of cadaverine was detected by HPLC (Fig. 5d, e), which was the first biosynthesis of cadaverine in *O. polymorpha*. Similarly, the copy numbers of the *HSA* gene in different integrants arranged from 2.48 ± 0.42 to 10.24 ± 1.26 (Fig. 5f). The maximum level of HSA was detected to be 97.09 ± 2.45 mg/L in the OP031 strain harboring 10.24 ± 1.26 copies of *HSA* (Fig. 5g). Taken together, the CMGE-MC was a useful and efficient tool for multi-copy integration in *O. polymorpha*.

### Multi-copy integration in *Saccharomyces cerevisiae*

**Fig. 6:**
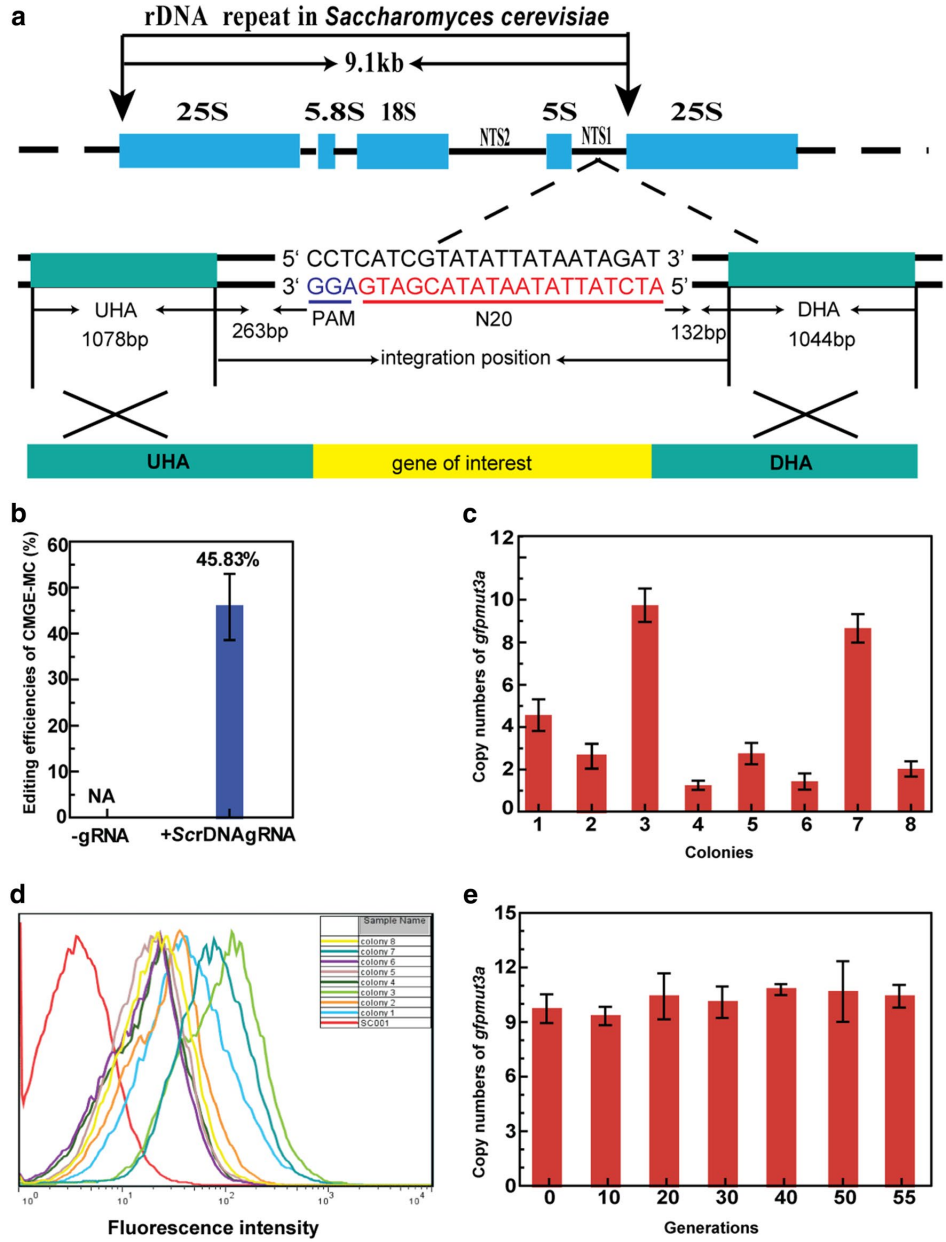
Multi-copy integration of *gfpmut3a* at rDNA cluster in *S. cerevisiae.* **a** Precise integration site of *gfpmut3a* at rDNA cluster. **b** Integration efficiencies of *gfpmut3a* at the rDNA repeats with and without the expression of targeting gRNA. **c** Copy numbers of *gfpmut3a* in eight randomly selected colonies. **d** Flow cytometry analysis of the expression of *gfpmut3a* in eight randomly selected colonies. **e** Stability of multi-copy integration at rDNA repeats in the mutant SC007 (SC001 rDNA::*gfpmut3a*)

To assess the effectiveness of the multi-copy integration method in other organism, *S. cerevisiae* that is one of the most intensively studied eukaryotic model organisms was selected. The gRNA delivery vector pWYE3225 and the *gfpmut3a* expression cassette flanked by up- and downstream homologous arms were co-transformed into the strain SC006 (SC001/pWYE3224) constitutively expressing Cas9 protein. As a result, the desired integrants were obtained with the editing efficiency of 45.83 ± 7.22% (Fig. 6a, b and Additional file 1: Figure S18). Eight colonies were randomly selected to detect  copy numbers of *gfpmut3a* gene. As shown in Fig. 6c, the copy numbers were arranged from 1.25 ± 0.22 (colony 4) to 9.74 ± 0.79 (colony 3). Furthermore, the fluorescence intensity had an increased tendency with the increase of copy number of *gfpmut3a* gene (Fig. 6d). Therefore, the multi-copy integration method was effective in *S. cerevisiae.*

To evaluate the stability of multi-copy genes, the strain SC007 harboring 9.74 ± 0.79 copies of *gfpmut3a* was cultured in YPD medium without any selection force. The copy numbers of the *gfpmut3a* were constant over 96-h cultivation (Additional file 1: Figure S19). In addition, the strain SC007 was cultured in YPD medium without any selective pressure for 18.5 days (55 generations). As shown in Fig. 6e, copy numbers of the *gfpmut3a* were constant after long-term propagation. The results demonstrated that multi-copy genes were highly stable in *S. cerevisiae*, suggesting a possibly broad applicability of this method in various yeasts, in which there are rDNA repeats and the CRISPR–Cas9 can work efficiently.

## Discussion

Genetic manipulation technologies, especially multiplex genome editing methods, are playing an important role in understanding gene function and developing rational design for biological engineering [22, 27, 33]. Recently, the CRISPR–Cas9 system has been employed as efficient genome editing tools in bacteria, fungi and higher eukaryotes [34–36]. In this study, we developed a CRISPR–Cas9-assisted multiplex genome editing method in yeasts.

The CRISPR–Cas9 system has enabled genome editing in different yeasts (Additional file 1: Table S6) [23, 34, 37]. Although CRISPR–Cas9-mediated genome editing methods have been developed in *O. polymorpha* by Numamoto et al. and Juergens et al. [17, 24], these methods only adapt for a single gene deletion and disruption (Additional file 1: Table S6). In this study, the CRISPR–Cas9-assisted multiplex genome editing method was developed for replacement, point mutation, multiple simultaneous knock-outs, multi-locus and multi-copy integrations of target genes in *O. polymorpha* and multi-copy gene integration in *S. cerevisiae* (Additional file 1: Table S6).

Furthermore, our study for the first time achieved CRISPR–Cas9-mediated markerless multi-copy integration in yeasts using rDNA repeats as the integration loci (Additional file 1: Table S6). Although rDNA repeats have been used extensively to integrate foreign genes in yeasts, selectable markers were required and multi-copy markers would be integrated into the chromosomes, which might interfere with the subsequent manipulation [28, 38]. In *S. cerevisiae*, *Ty* elements (the transposable elements) were used as the integration loci to integrate markerless multi-copy genes [33]. However, not all yeast strains have *Ty* elements, and the copy number and distribution on the genome of *Ty* elements also varied dramatically in different strains [28, 39, 40], suggesting that the available method was not applicable to the strains whose genetic background was not clear. In contrast, the copy numbers of rDNA repeats are highly constant in the same yeast species [28, 38]. Therefore, the multi-copy integration method developed in this study has broad practicability in different yeast stains.

Moreover, our method was efficient for various genetic manipulations in yeasts. To compare the editing efficiency of our method and pervious Cas9-based methods, the same gene *OpADE2* was used for gene deletion in *O. polymorpha*. As a result, the editing efficiency for the deletion of *OpADE2* was 62.18 ± 6.17% (Fig. 2b and Additional file 1: Figure S5). However, editing efficiencies of *OpADE12* (*OpADE2,* note S2) deletion in Numamoto’s and Juergens’s methods were 47% and 9%, respectively. In addition, editing efficiencies of different gene disruptions were 17–71% in Numamoto’s method, whereas those of gene deletion, integration, precise point mutation, multiplex genes knock-outs, multi-locus and multi-copy integration were 23.61–75.00% in our study (Additional file 1: Table S6).

Recently, more and more bio-products have been synthesized in microorganisms, which usually require the integration of multiple genes into the host’s chromosome [41]. In spite of rapid development of genetic engineering, integrations of multiple genes into the genome are still laborious and time consuming. In order to be convenient for constructing the engineering strain, it is necessary for the development of the multiplex gene integration method [37, 42]. The method using rDNA cluster as the target site can mediate simultaneous integration of multiple genes in *O. polymorpha* [27]. However, using this method, many copies of the marker gene *OpURA3* would be left in the genome, which might interfere with the subsequent manipulation. In addition, the editing efficiency of this method was relatively low (only 1/11). In this study, the CMGE-ML can simultaneously mediate markerless integrations of three foreign genes at three loci located in three different chromosomes with the editing efficiency of 30.56 ± 2.40%, suggesting that CMGE-ML was an effective tool in *O. polymorpha*.

High expression of gene is usually required to generate a detectable phenotype or obtain high productions of target products [43, 44]. The integration of multi-copy target gene into the genome is an important method for increasing gene expression, especially in the organism without episomal plasmids [28, 45]. In *O. polymorpha*, traditional methods for multi-copy integration include autonomously replicating plasmids-mediated random integration and recombinant plasmid-mediated site-specific integration [14, 46, 47]. Using the random integration method, up to 100 copies of target genes could be integrated at random sites, and using the site-specific integration method, 2–40 copies of target genes could be integrated at specific sites [14, 47]. However, all available multi-copy integration methods in *O. polymorpha* required selectable markers, and the whole vector containing the undesired DNA fragment would be integrated into the chromosome. Consequently, many copies of marker genes and other undesirable genetic elements (e.g., replication origin, plasmid-borne and bacterial antibiotic resistance genes) were left in the chromosome. In this study, editing templates for multi-copy integrations did not contain any antibiotic resistance cassette. Therefore, multi-copy engineered strains not containing any antibiotic resistance cassette would be stable in industrial application. Moreover, ~ 10 copies of the fusion expression cassette *P*_*ScTEF1*_-*TAL*-*P*_*ScTPI1*_-*4CL*-*P*_*ScTEF2*_-*STS* were integrated into the rDNA cluster via CMGE-MC by one step, achieving a ~ 20-fold increase of the resveratrol production, indicating that the method was a simple and powerful tool. In addition, the successful biosynthesis of cadaverine and HSA suggested that CMGE-MC had broad practicability.

The CRISPR–Cas9-assisted genome editing system in this study was established by expression of Cas9 and transcription of gRNAs in the chromosome with following advantages: (i) The expressions of Cas9 in the chromosome were more stable than the episomal plasmid which was potentially unstable in *O. polymorpha* (Note S1) and its copy numbers were variable in different cells. (ii) It was convenient for iterative genome editing without the need for construction and curing of the Cas9 expression plasmid every time. In addition, the expression of chromosome-borne Cas9 maintained at the relative low level of Cas9 protein, which might reduce the toxic impact of Cas9 protein on cell growth and metabolism [48–50]. In contrast, plasmid-based CRISPR–Cas9 methods might impose cells a fitness burden to maintain multi-copy plasmids [35].

## Conclusions

The CMGE developed in this study is effective for markerless genome editing in yeasts. In particular, multiplex gene knock-outs, CMGE-ML and CMGE-MC were powerful tools in fundamental and applied researches. In addition, the successful application of CMGE-MC in *S. cerevisiae* suggests that it might be applicable to a wide range of yeasts.

## Methods

### Strains, primers and genes

The strains and plasmids used in this study are listed in Additional file 1: Table S1. *E. coli* EC135 lacking all known restriction-modification (R-M) systems and orphan DNA methyltransferases (MTases) was used as the cloning host [51]. The primer synthesis (Additional file 1: Table S2) and DNA sequencing were performed by Invitrogen company (Shanghai, China) and Beijing Genomics Institute (BGI, Beijing, China), respectively. The *cadA* gene encoding lysine decarboxylase from *E. coli*, *TAL* gene encoding tyrosine ammonia-lyase from *Herpetosiphon aurantiacus*, *4CL* gene encoding 4-coumaryl-CoA ligase from *Arabidopsis thaliana*, *STS* gene encoding stilbene synthase from *Vitis vinifera*, and human serum albumin gene *HSA* were all synthesized by GenScript (Nanjing, China) in codon-optimized versions for expression in *O. polymorpha*.

### Culture and growth conditions

*Escherichia coli* cells were grown at 37 °C in Luria–Bertani (LB) medium (10 g/L tryptone, 5 g/L yeast extract and 10 g/L NaCl). *O. polymorpha* cells were grown at 37 °C in YPD medium (10 g/L yeast extract, 20 g/L peptone and 20 g/L glucose). To induce the expression of Cas9 protein that was controlled by the inducible promoter *P*_*OpMOX*_ in *O. polymorpha*, YPM medium (10 g/L yeast extract, 20 g/L peptone and 5 mL/L methanol) was used [15]. *S. cerevisiae* was grown at 30 °C in YPD medium before transformation. After transformation, cells were grown in appropriate synthetic complete (SC) medium minus the auxotrophic compound (FunGenome Company, Beijing, China) complemented by the plasmids. To induce the expression of Cas9 protein that was controlled by the inducible promoter *P*_*ScGAL1*_ in *S. cerevisiae*, cells were grown in SC medium with 2% galactose but without uracil media until the OD_600_ reached 0.5 [52]. When necessary, ampicillin (100 μg/mL for *E. coli*), kanamycin (50 μg/mL for *E. coli*), zeocin (25 μg/mL for *E. coli* or 100 μg/mL for *O. polymorpha*) and G418 (100 μg/mL for *O. polymorpha*) were added to the medium.

### Preparation of competent cell and transformation

The preparation of electro-competent cells and DNA transformation of *O. polymorpha* were performed following the procedure described by Faber et al. [53]. DNA transformation of *S. cerevisiae* was carried out using LiAc/SS carrier DNA/PEG method [54, 55]. Approximately, 1 μg of plasmid DNA (or 1 μg of gRNA delivery plasmid and 3 μg of editing template) was used per transformation.

### Construction of plasmids and editing templates

For constitutive expression of Cas9 protein in *O. polymorpha*, the DNA fragment containing the *Cas9* gene and the promoter *P*_*ScTEF1*_ was PCR amplified from the plasmid pWYE3202 (pCRCT Addgene plasmid # 60621). Up- and downstream homologous arms (~ 1.5 kb) of the *OpMET2* gene (*OpMET2*-*UHA*-*DHA*) were amplified from the genomic DNA of *O. polymorpha*. Three PCR fragments were then Gibson assembled into the *Bgl*II/*Xba*I site of pWYE3200 to generate the plasmid pWYE3208 (pWYE3200-*P*_*ScTEF1*_-*Cas9*-*OpMET2*-*UHA*-*DHA*) [15]. Subsequently, the plasmid pWYE3216 (pWYE3200-*P*_*OpMOX*_-*Cas9*-*OpMET2*-*UHA*-*DHA*) harboring the methanol-inducible promoter *P*_*OpMOX*_ from *O. polymorpha*, the Cas9 gene and the *OpMET2*-*UHA*-*DHA* was constructed in a similar manner. To construct the gRNA delivery vector, the *OpADE2*-*UHA* ( ~ 1.5 kb)-*DHA* ( ~ 1.5 kb) from *O. polymorpha*, the promoter *P*_*ScSNR52*_ from *S. cerevisiae*, and the synthesized crRNA, 20-bp complementary region (N_20_) and *ScSUP4t* were assembled into the *Bgl*II/*Bam*HI site of pWYE3201 (pWYE3200 derivative, the zeocin resistance gene *zeo*^*R*^ was replaced by the G418 resistance gene *G418*^*R)*^ to generate pWYEN (a generic term of all gRNA delivery vector and “N” represents the serial number).

Three gRNA expression cassettes targeting *OpLEU2*, *OpURA3* and *OpHIS3* genes were constructed into the vector pWYE3215 (pWYE3201- *OpLEU2*gRNA-*OpURA3*gRNA-*OpHIS3*gRNA-*OpADE2*-*UHA*-*DHA*) for multiplex genome engineering. The gRNA expression cassettes for *OpHIS3* and *OpLEU2* were PCR amplified from the vectors pWYE3213 (pWYE3201-*OpHIS3*gRNA-*OpADE2*-*UHA*-*DHA*) and pWYE3209 (pWYE3201-*OpLEU2*gRNA-*OpADE2*-*UHA*-*DHA*), respectively. The two expression cassettes were Gibson assembled into the plasmid pWYE3212 (pWYE3201-*OpURA3*gRNA-*OpADE2*-*UHA*-*DHA*) linearized with *Bam*HI to generate the plasmid pWYE3215.

In *S. cerevisiae*, the expression of Cas9 protein was controlled by the inducible promoter *P*_*ScGAL1*_. The *Cas9* gene fragment was Gibson assembled into the vector pWYE3222 (pYES2.0/CT, Invitrogen) linearized with *Bam*HI and *Eco*RI to generate the plasmid pWYE3224 (pWYE3222-*P*_*ScGAL1*_-*Cas9*). The rDNA-gRNA delivery vector pWYE3225 (pWYE3223-rDNAgRNA) was constructed by ligating the gRNA expression elements into the vector pWYE3223 (pESC-leu2, Addgene plasmid #20120).

For gene deletion or precise point mutation, the UHA and DHA (~ 1 kb) of the target gene (or of the cut site) were amplified from *O. polymorpha* genomic DNA and jointed by Splicing Overlapping Extension (SOE) PCR. For gene integration, the editing template was PCR amplified from the donor plasmid that was constructed by Gibson assembling the UHA and DHA (~ 1 kb) of the target gene, the promoter *P*_*ScTEF1*_ and the desired gene with a synthetic terminator into the vector pWYE3200 (see Additional file 1 for details).

### Editing efficiencies

The mutants were identified by cell growth phenotype and/or PCR amplification. The editing efficiency was defined as the ratio of the desired mutants to the total tested colonies and calculated using the following formula: Editing efficiency = Number of desired mutants/Number of total tested colonies [58].

### Flow cytometry analysis

The *gfpmut3a* gene encoding a green fluorescent protein (GFP) with enhanced intense fluorescence was used as the reporter gene. Cells integrating the *gfpmut3a* gene were grown in YPD plate overnight and then inoculated into YPD media at a starting OD_663_ of 0.1 for *O. polymorpha* (or OD_600_ of 0.1 for *S. cerevisiae*). Cells were harvested after reached the exponential phase, and then washed and resuspended in phosphate-buffered saline (PBS) buffer. The GFP fluorescence intensities were measured by BD FACS CaliburTM flow cytometer equipped with an argon laser (emission at 488 nm and 15 mW) and a 525-nm band-pass filter. For each sample, 30,000 events were collected at a rate of 1000–2000 events per second. Cells without integrating the *gfpmut3a* gene were used as a control to determine the background fluorescence.

### Gene copy number estimation

Primers for qPCR are listed in Additional file 1: Table S2. Genomic DNA was extracted using the TIANamp Yeast DNA Kit (Tiangen, China) according to the manufacturer’s protocol. Gene copy numbers were determined by quantitative PCR (qPCR) as described by Kolacsek et al. [56]. The *OpMOX* and *ScALG9* genes were used as the references of *O. polymorpha* and *S. cerevisiae*, respectively. The plasmid pWYE3227 harboring partial sequences of *gfpmut3a*, *OpMOX* and *ScALG9* genes was used as the template for standard curves to estimate the copy number of *gfpmut3a*. Similarly, to estimate the copy number of *cadA*, *HSA* or *P*_*ScTEF1*_-*TAL*-*P*_*ScTPI1*_-*4CL*-*P*_*ScTEF2*_-*STS* expression cassette, the vector pWYE3228 harboring partial sequences of *OpMOX*, *cadA, HSA and TAL* was used for standard curves. Quantitative PCR was performed using GoTaq qPCR master mix (Promega, USA) in a 20-μL mixture with a LightCycler^®^ 96 Real-Time PCR System (Roche, Switzerland).

### Stability detection

To evaluate the stability of multi-copy genes, the integrants were cultivated in nonselective YPD medium by serial-subcultures and continuous culture, respectively. For continuous culture, transformants were cultivated in YPD medium for 96 h. 1 mL cell cultures were collected at the 24-h interval to extract the genomic DNA for estimation of the copy number.

To evaluate the stability of multi-copy integrants in a long-term lab evolution, integrants were cultivated in nonselective YPD medium for 55 generations (18.5 days). Mutants were inoculated into a 500 mL shake-flask containing 50 mL YPD medium. 2 mL culture broth was transferred to 50 mL fresh YPD medium every 8 h and this procedure was repeated fifty-four times. 2 mL cell cultures were collected every 10 generations to extract the genomic DNA for estimation of the copy number.

### Potential off-target sites predict by CAS-OFFinder

In the CAS-OFFinder program, there are three key parameters affecting the prediction results: “Mismatch Number”, “DNA Bulge Size”, “RNA Bulge Size”. The “Mismatch Number” can be set as 0–9. “DNA Bulge Size” and “RNA Bulge Size” can be set as 0–2. We finally set the “Mismatch Number” as 3, the “DNA Bulge Size” and “RNA Bulge Size” both as 2 based on these reasons: (i) as the number of mismatches increases, the total number of potential off-target sites dramatically increases as well. So if set the “Mismatch Number” as 9 and sequencing all the potential off-target sites one by one is an almost impossible task. (ii) In general, two mismatches, particularly those occurring in a PAM proximal region, considerably reduced SpCas9 activity. Furthermore, three or more mismatches eliminated detectable SpCas9 cleavage in most loci [57].

### Fermentation in shake flasks

*Ogataea polymorpha* cells were cultured in 500 mL shake flasks containing 50 mL YPD media at 37 °C with shaking at 200 rpm. For production of resveratrol, the media were supplemented with 5 mM tyrosine as precursor. Similarly, 50 mM lysine was added to the media to produce cadaverine. Throughout the time course of the experiment, cell cultures were collected at the 12-h interval to detect the OD_663_ and the concentration of resveratrol or cadaverine in the fermentation samples. The wild-type *O. polymorpha* OP001 was used as a negative control. All experiments and measurements were performed at least in triplicate.

### Analysis of product concentration

The concentration of resveratrol was quantified on HPLC (Agilent) equipped with an Eclipse XDB-C18 column (4.6 × 150 mm; Agilent Technologies, USA). The eluent flow was at a constant rate of 1.0 mL/min with 70% reagent A (0.1% phosphoric acid v/v) and 30% reagent B (acetonitrile) [32]. Detection wavelength was set at 304 nm and the column was maintained at 40 °C. The concentration of cadaverine was determined by HPLC as described by Wu et al. [58]. The concentrations of resveratrol and cadaverine from the fermentation samples were calculated from the standard curves using the standards from Sigma, USA. The commercially available QuantiChrom BCG Albumin Assay Kit (DIAG-250) was used to determine the concentration of HSA in culture medium.

## Additional file

**Additional file 1: Table S1.** Strains and plasmids used in this study. **Table S2.** Primers used in this study. **Table S3.** Potential off-target sites of CRISPR–Cas9 mediated point mutation in the gene *OpURA3*. **Table S4.** Editing efficiencies mediated by CRISPR–Cas9 in *O. polymorpha*. **Table S5.** Editing efficiencies mediated by endogenous HRS in *O. polymorpha.* **Table S6.** Editing efficiencies of CRISPR–Cas9-assisted genome engineering methods in different yeasts. **Figure S1.** Analysis of the editing efficiency mediated by endogenous HRS at the *OpADE2* site by cell growth phenotype on YPD and SC without adenine (SC-ADE) plates. **Figure S2.** PCR identifications of the deletion of *OpLEU2* (A) and *OpURA3* (B) genes, respectively. **Figure S3.** Evictions of the linearized gRNA delivery vector and the linearized Cas9 protein expression vector after gene editing. **Figure S4.** Verification of gene deletions by auxotrophic phenotype analysis. **Figure S5.** Effect of HA (homologous arm) on editing efficiency of CRISPR–Cas9 mediated gene deletion in *O. polymorpha*. **Figure S6.** PCR identifications of simultaneous deletions of genes *OpLEU2*, *OpHIS3* and *OpURA3*. **Figure S7.** Verification of multiplex knock-outs by auxotrophic phenotype analysis. **Figure S8.** The identification of the point mutation by cell growth phenotype. The YPD plate and SC plate without uracil (SC-URA). **Figure S9.** Verifications of point mutation of the gene *OpURA3.* **Figure S10.** DNA sequencing of similar genomic loci of point mutation site in the gene *OpURA3* of the mutant OP040 (OP001 *OpURA3*^G73T^). **Figure S11.** PCR identifications of *gfpmut3a* expression cassette separately integrated at *OpLEU2* (A), *OpHIS3* (B) and *OpURA3* (C) loci. **Figure S12.** Analysis of editing efficiencies mediated by endogenous homologous recombination system at the gene *OpLEU2* (A), *OpHIS3* (B) and *OpURA3* (C) sites by cell growth phenotype. **Figure S13.** Editing efficiencies at three gene sites by two different methods. **Figure S14.** PCR identifications of simultaneously multi-loci genomic integration. **Figure S15.** PCR identification of *gfpmut3a* expression cassette by multi-copy integration at rDNA cluster in *O. polymorpha*. **Figure S16.** Flow cytometry analysis of the expression of *gfpmut3a* in eight randomly selected *O. polymorpha* colonies. **Figure S17.** Stability of multi-copy integration of *gfpmut3a* at rDNA repeats in the mutant OP025 upon continuous culture for 96 h. **Figure S18.** PCR identification of *gfpmut3a* expression cassette by multi-copy integration at rDNA cluster in *S. cerevisiae*. **Figure S19.** Stability of multi-copy integration of *gfpmut3a* at rDNA repeats in the mutant SC007 upon continuous culture for 96 h.

## Abbreviations

*O. polymorpha*: *Ogataea polymorpha*
*S. cerevisiae*: *Saccharomyces cerevisiae*
HPLC: high-performance liquid chromatography
OD_600_: optical density at wavelength (λ) 600 nm
OD_663_: optical density at wavelength (λ) 663 nm
WT: wild type
OP: *Ogataea polymorpha*
HA: homologous arm
DHA: downstream homologous arm
UHA: upstream homologous arm
