# Supplementary material for: Efficient CRISPR–Cas9 mediated multiplex genome editing in yeasts
Source: Biotechnol Biofuels. 2018 Oct 10;11:277. doi: 10.1186/s13068-018-1271-0 (PMC6180501; doi:10.1186/s13068-018-1271-0)
Supplement: Supplementary file 1 — Additional file 1: Table S1. Strains and plasmids used in this study. Table S2. Primers used in this study. Table S3. Potential off-target sites of CRISPR–Cas9 mediated point mutation in the gene OpURA3. Table S4. Editing efficiencies mediated by CRISPR–Cas9 in O. polymorpha. Table S5. Editing efficiencies mediated by endogenous HRS in O. polymorpha. Table S6. Editing efficiencies of CRISPR–Cas9-assisted genome engineering methods in different yeasts. Figure S1. Analysis of the editing efficiency mediated by endogenous HRS at the OpADE2 site by cell growth phenotype on YPD and SC without adenine (SC-ADE) plates. Figure S2. PCR identifications of the deletion of OpLEU2 (A) and OpURA3 (B) genes, respectively. Figure S3. Evictions of the linearized gRNA delivery vector and the linearized Cas9 protein expression vector after gene editing. Figure S4. Verification of gene deletions by auxotrophic phenotype analysis. Figure S5. Effect of HA (homologous arm) on editing efficiency of CRISPR–Cas9 mediated gene deletion in O. polymorpha. Figure S6. PCR identifications of simultaneous deletions of genes OpLEU2, OpHIS3 and OpURA3. Figure S7. Verification of multiplex knock-outs by auxotrophic phenotype analysis. Figure S8. The identification of the point mutation by cell growth phenotype. The YPD plate and SC plate without uracil (SC-URA). Figure S9. Verifications of point mutation of the gene OpURA3. Figure S10. DNA sequencing of similar genomic loci of point mutation site in the gene OpURA3 of the mutant OP040 (OP001 OpURA3G73T). Figure S11. PCR identifications of gfpmut3a expression cassette separately integrated at OpLEU2 (A), OpHIS3 (B) and OpURA3 (C) loci. Figure S12. Analysis of editing efficiencies mediated by endogenous homologous recombination system at the gene OpLEU2 (A), OpHIS3 (B) and OpURA3 (C) sites by cell growth phenotype. Figure S13. Editing efficiencies at three gene sites by two different methods. Figure S14. PCR identifications of simultaneously multi-loci [file 13068_2018_1271_MOESM1_ESM.docx]

**Additional file**

**Materials and Methods**

**Construction of plasmids for genome modification in *O. polymorpha*.**

For integrations of *gfpmut3a* at the gene *OpLEU2* site, the UHA and DHA (~1 kb) of *OpLEU2* were amplified from the genomic DNA of *O. polymorpha* and the expression cassette *P_ScTEF1_*-*gfpmut3a-OpAOXt was* amplified from the vector pBAD43-25. The three fragments were then Gibson assembled into the *Bgl*II/*Bam*HI site of pWYE3200 to generate the plasmid pWYE3210 (pWYE3200-*OpLEU2UHA-P_ScTEF1_*-*gfpmut3a-OpAOXt-DHA*). Similarly, for integrations of *gfpmut3a* at the *OpURA3*, *OpHIS3* and *Op*rDNA sites respectively, the plasmids pWYE3211 (pWYE3200-*OpURA3UHA-P_ScTEF1_*-*gfpmut3a-OpAOXt-DHA*), pWYE3212 (pWYE3200-*OpHIS3UHA-P_ScTEF1_*-*gfpmut3a-OpAOXt-DHA*) and pWYE3221 (pWYE3200-*Op*rDNA*UHA-P_ScTEF1_*-*gfpmut3a-OpAOXt-DHA*) were constructed. After constructions, the fragments UHA-*P_ScTEF1_*-*gfpmut3a*-OpAOXt-DHA for integration at each site were PCR amplified from the vectors respectively as the editing templates.

To integrate *STS* at the gene *OpLEU2* site，the UHA and DHA (~1 kb) of *OpLEU2* and the expression cassette *P_ScTEF2_*-*STS-*T_Guo1_ were Gibson assembled into the *Bgl*II/*BamH*I site of pWYE3200 to generate the plasmid pWYE3216 (pWYE3200-*OpLEU2UHA-P_ScTEF1_*-*STS-*T_Guo1_*-DHA*). Similarly, for integrations of *TAL* , *4CL* at the *URA3*, *HIS3* respectively，the plasmids pWYE3217 (pWYE3200-*OpURA3UHA-P_ScTEF1_*-*TAL-*T_Guo1_*-DHA*) and pWYE3218 (pWYE3200-*OpHIS3UHA-P_ScTPI1_*-*4CL-*T_Guo1_*-DHA*) were constructed. After constructions, the fragments *UHA-expression cassette-DHA* for integration at each site were PCR amplified from the vectors respectively as the editing templates.

For multi-copy integrations of the fusion expression cassette *P_ScTEF1_*-*TAL-P_ScTPI1_-4CL-P_ScTEF2_-STS,* the UHA and DHA (~1 kb) of *Op*rDNA and the fusion expression cassette were Gibson assembled into the *Bgl*II/*BamH*I site of pWYE3200 to generate the plasmid pWYE3230 (pWYE3200-*Op*rDNA*UHA- P_ScTEF1_*-*TAL-P_ScTPI1_-4CL-P_ScTEF2_-STS-DHA*). Similarly, to multi-copy integrate *HSA* and *cadA* the plasmids pWYE3231 (pWYE3200-*Op*rDNA*UHA-P_ScTEF1_*-*cadA-DHA*)*P_ScTEF1_*-*HSA-*T_Guo1_ and pWYE3232 (pWYE3200-*Op*rDNA*UHA-P_ScTEF1_*-*HSA-DHA*) were constructed. After constructions, the fragments *Op*rDNA*UHA-expression cassette-DHA* for integrations were PCR amplified from the vectors respectively as the editing templates.

For deletion of gene *OpADE2*, the *OpURA3* was selected as the integration locus of gRNA expression cassette. To construct the gRNA delivery vector, the *OpURA3-UHA* (~1.5 kb)*-DHA* (~1.5 kb) from *O. polymorpha*, the promoter *P_ScSNR52_* from *S. cerevisiae*, and the synthesized crRNA, 20-bp complementary region (N_20_) and *ScSUP4t* were assembled into the *Bgl*II/*Bam*HI site of pWYE3201 to generate pWYE3233 (pWYE3201 derivative, G418^R^, *OpURA3*upHA-*P_ScSNR52_-OpADE2*gRNA-*OpURA3*downHA). *OpADE2* UHA and DHA of different lengths were amplified from *O. polymorpha* genomic DNA and jointed by Splicing Overlapping Extension (SOE) PCR as editing templates. The knockout procedure was similar to the description in Figure 1B.

For multiple simultaneous knock-outs, three genes *OpURA3，OpHIS3* and *OpLEU2* were selected as the target loci. The UHA and DHA (~1 kb) fragments of each target gene were jointed by Splicing Overlapping Extension PCR as editing template. Three different editing templates for deletions of *OpURA3*, *OpHIS3 and OpLEU2* were co-transformed into the strain OP009 with the linearized vector pWYE3215 harboring three gRNAs expression cassettes to separately target three sites.

**Note S1**

The stability of episomal plasmid in *O. polymorpha* is still contentious*.* Although recently the plasmid-based CRISPR-Cas9 systems have been established in *O. polymorpha [*[*1*](#_ENREF_1)*,* [*2*](#_ENREF_2)*]*, there have been some studies suggesting that no stable extrachromosomally replicating plasmids are available for *H. polymorpha [*[*3*](#_ENREF_3)*,* [*4*](#_ENREF_4)*].* Plasmids containing autonomously replicating sequences (ARS) have been isolated and demonstrate episomal replication *O*. *polymorpha [*[*5*](#_ENREF_5)*].* However, none of these extrachromosomally replicating plasmids are stable enough for general use [[3](#_ENREF_3)]. This is mainly because circular plasmids even one with an ARS and limited homology to the nuclear DNA, showed a very low mitotic stability of less than 5% for 10 generations [[6](#_ENREF_6)] and easily integrate into the genome with high variations in copy number particularly in cultivation under selective conditions [[7](#_ENREF_7), [8](#_ENREF_8)]. Although Juergens et al used a plasmid named pUDP046 with a pangenomic yeast replication origin panARS to express Cas9 and gRNAs，they had to sequence the genome to verify that no pUDP046 plasmid sequences had recombined into the chromosomal DNA after genome editing, which was costly and time-consuming [[2](#_ENREF_2)].

**Note S2**

The *ADE2* gene in *Saccharomyces cerevisiae* (*ScADE2*) encodes phosphoribosylaminoimidazole carboxylase involved in adenine biosynthesis. Loss-of-function mutations in *ScADE2* result in a red phenotype due to accumulation of the oxidized form of 5-amino imidazole ribonucleotide [[9](#_ENREF_9)]. Its homolog gene in *O*. *polymorpha* was named *OpADE2* in our study, which was consistent with previous study [[2](#_ENREF_2), [10](#_ENREF_10)]. However, in Numamoto's study the homolog of *ScADE2* was named *OpADE12.* In addition, through sequence alignment we found the nucleotide sequence of *OpADE12*_gRNA (GCTTGAAACCCCACACGCGT) in Numamoto's study was included in the gene *OpADE2* in our study (Sequence S1). Therefore, the gene *OpADE12* in Numamoto's study and the gene *OpADE2* in our study was the same gene.

Sequence S1

*OpADE2* ATGGACTCAAAGGTCGTTGGAATTTTGGGCGGCGGCCAGCTCGGCCGCATGATGGTCGAGGCAGCCAGCCGGCTGAATATCAAGACAGTGATTCTTGAGAACGGTGCAGATTCACCGGCCAAGCAGATCAATTCCAGTACAGAACACATCGACGGCTCCTTCAACGATGAGGCGGCCATCCGCAAGCTCGCGGAAAAATGCAACGTGCTGACCGTCGAGATTGAGCACGTTGATGTTGAGGCCTTGAAGAAAGTGCAGGAGCAGACTTCCGTCAAGATTTATCCATCTCCTGAGACCATTGCTCTTATCAAGGACAAATACTTGCAAAAAGAGCATCTGATCAGAAACCAGATCGCGGTTGCCGAGTCCACTGCTGTTGAAAGCACTTCAGGAGCCTTGCAATCTGTGGGACAGAAGTATGGATACCCGTACATGCTCAAGTCCAGAACGATGGCTTATGACGGTAGGGGTAACTTTGTTGTTGAGGACGATTCCAAGATCCCAGAGGCTTTGGAGGCCTTGAAGGACAGACCGTTATATGCTGAAAAATGGGCTCCTTTCACCAAGGAGCTAGCAGTGATGGTGGTTCGGGGTCTTGGCGGAGACGTCCATGCCTACCCAACCGTAGAGACTATTCACAAAAACAATATCTGCCACACAGTGTTTGCACCTGCGCGTGTCAATGACACCATACAGAAGCGCGCGCAACTCCTGGCAGAGAAGGCTGTGTCTGCATTTTCGGGAGCAGGAATTTTTGGTGTCGAAATGTTCCTGCTTCCAAATGACGAGTTGTTGATCAACGAAATTGCCCCTAGACCGCACAACTCTGGACATTACACTATCGACGCGTGCGTGACGAGCCAGTTTGAGGCCCACATCCGTGCCGTTTGCAGTCTGCCGCTACCAAAGAACTTTACTTCTCTATCCACACCATCTACCCATGCTATCATGCTAAACGTGTTGGGTAGCTCTAACCCAGAAGAATGGTTGCAAAAGTGCAAGAGAGCGCTTGAAACCCCACACGCGTCGGTTTACCTGTACGGAAAATCCAACAGACCGGGCCGGAAACTGGGTCACATCAACATTGTCTCCCAGTCCATGGACGACTGCATCCGTCGTCTAGAGTACATAGACGGCCAATCCGACACACTGAAAGAGCCTAAAGACAACATACATGTTGCAGGAACTAGCAGCAAACCGCTCGTCGGCGTGATAATGGGCTCAGACTCGGATCTGCCTGTGATGTCCGTTGGTTGCAATATTTTAAAGGCTTTTGGTGTTCCTTTCGAGGTTACCATTGTGTCTGCCCACAGAACGCCTCAGAGAATGGTCAAGTACGCTGCCGAAGCCCCAGAGAGGGGAATACGGTGCATCATCGCTGGTGCTGGGGGAGCTGCCCATCTACCAGGAATGGTTGCTGCCATGACTCCATTGCCGGTCATTGGTGTTCCCGTCAAGGGATCGACTCTCGACGGAGTCGACTCGCTGTATTCGATAGTTCAGATGCCAAGAGGAGTGCCTGTGGCCACTGTTGCCATCAACAATGCCACCAACGCTGCGCTTCTGGCCGTGCGTATTCTTGGCTCGTCCGACCCCGTGTATTTCAGCAAGATGGCTAAATACATGAGCGAGATGGAGAATGAGGTTCTTGAAAAAGCTGAACGACTGGGCTCTGTTGGCTATGAGGAATACCTTAACAAATAG

*OpADE12*_gRNA

**Table S1. Strains and plasmids used in this study**

| **Strain or Plasmid** | **Characteristics** | **Reference/**  **Source** |
| --- | --- | --- |
| **Strain**  *Escherichia coli* EC135 | F-*mcrA*Δ(*mrr*-*hsdRMS*-*mcrBC*)φ80*lacZ*Δ*M15*Δ*lacX74araD*139Δ(*ara*-*leu*)7697 *galUgalKrpsL*  e*ndA1nupG*Δ*dcm*::*FRT* *recA*+Δ*dam*::*FRT*, genotype of R-M systems: *mcrA*Δ(*mrr*-*hsdRMS*-*mcrBC*)  Δ*dcm*::*FRT*Δ*dam*::*FRT* | [[11](#_ENREF_11)] |
| *Ogataea polymorpha* |  |  |
| CGMCC7.89(OP001) | Wild type | Laboratory  strain |
| OP009 | Zeo^R^, OP001Δ*OpMET2*::*P_ScTEF1_-Cas9* | This study |
| OP010 | Zeo^R^, G418^R^, OP001Δ*OpMET2*::*P_ScTEF1_-Cas9*  Δ*OpADE2*::*OpLEU2*gRNA Δ*OpLEU2*::*gfpmut3a* | This study |
| OP011 | Zeo^R^, OP001Δ*OpMET2*::*P_ScTEF1_-Cas9*  Δ*OpLEU2*::*gfpmut3a* | This study |
| OP012 | OP001Δ*OpLEU2*::*gfpmut3a* | This study |
| OP013 | Zeo^R^, G418^R^, OP001Δ*OpMET2*::*P_ScTEF1_-Cas9*  Δ*OpADE2*::*OpURA3*gRNA Δ*OpURA3*::*gfpmut3a* | This study |
| OP014 | Zeo^R^, OP001Δ*OpMET2*::*P_ScTEF1_-Cas9*  Δ*OpURA3*::*gfpmut3a* | This study |
| OP015 | OP001Δ*OpURA3*::*gfpmut3a* | This study |
| OP016 | Zeo^R^, G418^R^, OP001Δ*OpMET2*::*P_ScTEF1_-Cas9*  Δ*OpADE2*::*OpHIS3*gRNA Δ*OpHIS3*::*gfpmut3a* | This study |
| OP017 | Zeo^R^, OP001Δ*OpMET2*::*P_ScTEF1_-Cas9*  Δ*OpHIS3*::*gfpmut3a* | This study |
| OP018 | OP001Δ*OpHIS3*::*gfpmut3a* | This study |
| OP019 | Zeo^R^, G418^R^, OP001Δ*OpMET2*::*P_ScTEF1_-Cas9*  Δ*OpADE2*::*OpHIS3*gRNA*-OpURA3*gRNA*-OpLEU2*gRNA  Δ*OpHIS3*::*4CL* Δ*OpURA3*::*TAL* Δ*OpLEU2*::*STS* | This study |
| OP020 | Zeo^R^, OP001Δ*OpMET2*::*P_ScTEF1_-Cas9*  Δ*OpHIS3*::*4CL* Δ*OpURA3*::*TAL* Δ*OpLEU2*::*STS* | This study |
| OP021 | OP001Δ*OpHIS3*::*4CL* Δ*OpURA3*::*TAL* Δ*OpLEU2*::*STS* | This study |
| OP022 | Zeo^R^, OP001Δ*OpMET2*::*P_OpMOX_-Cas9* |  |
| OP023 | Zeo^R^, G418^R^, OP001Δ*OpMET2*::*P_OpMOX_-Cas9*  Δ*OpADE2*::rDNAgRNA rDNA::*gfpmut3a* | This study |
| OP024 | Zeo^R^, OP001Δ*OpMET2*::*P_OpMOX_-Cas9*  OP001rDNA::*gfpmut3a* | This study |
| OP025 | OP001rDNA::*gfpmut3a* | This study |
| OP026 | Zeo^R^, G418^R^, OP001Δ*OpMET2*::*P_OpMOX_-Cas9*  Δ*OpADE2*::*rDNA*gRNA rDNA::*cadA* | This study |
| OP027 | Zeo^R^, OP001Δ*OpMET2*::*P_OpMOX_-Cas9*  rDNA::*cadA* | This study |
| OP028 | OP001rDNA::*cadA* | This study |
| OP029 | Zeo^R^, G418^R^, OP001Δ*OpMET2*::*P_OpMOX_-Cas9*  Δ*OpADE2*::*rDNA*gRNA rDNA::*HSA* | This study |
| OP030 | Zeo^R^, OP001Δ*OpMET2*::*P_OpMOX_-Cas9*  rDNA::*HSA* | This study |
| OP031 | OP001rDNA::*HSA* | This study |
| OP032 | Zeo^R^, G418^R^, OP001Δ*OpMET2*::*P_ScTEF1_-Cas9*  Δ*OpADE2*::*OpLEU2*gRNA Δ*OpLEU2* | This study |
| OP033 | Zeo^R^, OP001Δ*OpMET2*::*P_ScTEF1_-Cas9*  Δ*OpLEU2* | This study |
| OP034 | OP001Δ*OpLEU2* | This study |
| OP035 | Zeo^R^, G418^R^, OP001Δ*OpMET2*::*P_ScTEF1_-Cas9*  Δ*OpADE2*::*OpURA3*gRNA Δ*OpURA3* | This study |
| OP036 | Zeo^R^, OP001Δ*OpMET2*::*P_ScTEF1_-Cas9*  Δ*OpURA3* | This study |
| OP037 | OP001Δ*OpURA3* | This study |
| OP038 | Zeo^R^, G418^R^, OP001Δ*OpMET2*::*P_ScTEF1_-Cas9*  Δ*OpADE2*::*OpURA3*gRNA *OpURA3*^G73T^ | This study |
| OP039 | Zeo^R^, OP001Δ*OpMET2*::*P_ScTEF1_-Cas9*  *OpURA3*^G73T^ | This study |
| OP040 | OP001*OpURA3*^G73T^ | This study |
| OP041 | Zeo^R^, G418^R^, OP001Δ*OpMET2*::*P_OpMOX_-Cas9*  Δ*OpADE2*::*rDNA*gRNA rDNA::*TAL-4CL-STS* | This study |
| OP042 | Zeo^R^, OP001Δ*OpMET2*::*P_OpMOX_-Cas9*  rDNA::*TAL-4CL-STS* | This study |
| OP043 | OP001rDNA::*TAL-4CL-STS* | This study |
| OP044 | Zeo^R^, G418^R^, OP001Δ*OpMET2*::*P_ScTEF1_-Cas9*  Δ*OpURA3*::*OpADE2*gRNA  Δ*OpADE2* | This study |
| OP045 | Zeo^R^, OP001Δ*OpMET2*::*P_ScTEF1_-Cas9*  OP001Δ*OpADE2* | This study |
| OP046 | OP001Δ*OpADE2* | This study |
| OP047 | Zeo^R^, G418^R^, OP001Δ*OpMET2*::*P_ScTEF1_-Cas9*  Δ*OpADE2*:: *OpHIS3*gRNA*-OpURA3*gRNA*-OpLEU2*gRNA  Δ*OpLEU2*Δ*OpHIS3*Δ*OpURA3* | This study |
| OP048 | Zeo^R^, OP001Δ*OpMET2*::*P_ScTEF1_-Cas9*  OP001Δ*OpLEU2*Δ*OpHIS3*Δ*OpURA3* | This study |
| OP049 | OP001Δ*OpLEU2*Δ*OpHIS3*Δ*OpURA3* | This study |
| *Saccharomyces cerevisiae* |  |  |
| SC001 | DAY414(*MATαhis3*Δ*200 trp1-901 leu2-3*, *-112 ade2 LYS2*::(*lexAop*)*_4_*-*OpHIS3* *OpURA3*::(*lexAop*)*_8_*-*lacZ GAL4* | [[12](#_ENREF_12)] |
| SC006 | SC001/pYES2.0CT-*ScGAL1*-Cas9 | This study |
| SC007 | SC001 rDNA::*gfpmut3a* | This study |
| **Plasmids** |  |  |
| pMD19 T | Cloning vector | TaKaRa |
| pBAD43-25 | pAD123 derivative, *gfpmut3a* | BGSC ^a^ |
| pWYE3200 | Zeo^R^, *O. polymorpha* integrative vector | a gift from Dr. Xiuping He[[13](#_ENREF_13)] |
| pWYE3201 | pWYE3200 derivative, G418^R^ | This study^b^ |
| pWYE3202 | Amp^R^，pCRCT，*P_ScTEF1_-Cas9* | (Addgene plasmid # 60621)[[14](#_ENREF_14)] |
| pWYE3208 | pWYE3200 derivative,  Zeo^R^, *OpMET2*upHA-*P_ScTEF1_-Cas9-OpMET2*downHA | This study |
| pWYE3209 | pWYE3201 derivative,  G418^R^, *OpADE2*upHA-*P_ScSNR52_-OpLEU2*gRNA-*OpADE2*downHA | This study |
| pWYE3210 | pWYE3200 derivative,  Zeo^R^, *OpLEU2*upHA-*P_ScTEF1_-gfpmut3a-OpLEU2*downHA | This study |
| pWYE3211 | pWYE3201 derivative,  G418^R^, *OpADE2*upHA-*P_ScSNR52_-OpURA3*gRNA^*^-*OpADE2*downHA | This study |
| pWYE3212 | pWYE3200 derivative,  Zeo^R^, *OpURA3*upHA-*P_ScTEF1_-gfpmut3a-OpURA3*downHA | This study |
| pWYE3213 | pWYE3201 derivative,  G418^R^, *OpADE2*upHA-*P_ScSNR52_-OpHIS3*gRNA-*OpADE2*downHA | This study |
| pWYE3214 | pWYE3200 derivative,  Zeo^R^, *OpHIS3*upHA-*P_ScTEF1_-gfpmut3a-OpHIS3*downHA | This study |
| pWYE3215 | pWYE3201 derivative,  G418^R^, *OpADE2*upHA-*OpLEU2*gRNA-*OpURA3*gRNA-*OpHIS3*gRNA-*OpADE2*downHA | This study |
| pWYE3216 | pWYE3200 derivative,  Zeo^R^, *OpLEU2*upHA-*P_ScTEF2_-STS-OpLEU2*downHA | This study |
| pWYE3217 | pWYE3200 derivative,  Zeo^R^, *OpURA3*upHA-*P_ScTEF1_-TAL-OpURA3*downHA | This study |
| pWYE3218 | pWYE3200 derivative,  Zeo^R^, *OpHIS3*upHA-*P_ScTPI1_-4CL-OpHIS3*downHA | This study |
| pWYE3219 | pWYE3200 derivative,  Zeo^R^, *OpMET2*upHA-*P_OpMOX_-Cas9-OpMET2*downHA | This study |
| pWYE3220 | pWYE3201 derivative,  G418^R^, *OpADE2*upHA-*P_ScSNR52_-*rDNAgRNA-*OpADE2*downHA | This study |
| pWYE3221 | pWYE3200 derivative,  Zeo^R^, rDNAupHA-*P_ScTEF1_-gfpmut3a-*rDNAdownHA | This study |
| pWYE3222 | pYES2.0/CT | [Invitrogen](https://www.baidu.com/baidu.php?url=QWRK00Klc7afi0pzWv07T_NFiq5gk_QIL2zftzwlyoq1bNQTmxguC_BY4wP2lnj0cpKSnK0rE8n8GJjbCkW9ECtFnnwub1IPUDedwuF1Zbker-yh6xjrAkjwHQUT5Px8QYtLPNhJVqdMVSGlExy08JIRzZkWHu0K-XY2FXIiXoKHxgUqq6.7D_imLqap5bMlD1FWCRDsdXAjWYe_bD5_eGSW-WknUPMN5YTVZCmLUQ2SPXiEI8LIROwzs1Ij7xZkLq5H_erPXOJMlrgZwml3FJQ7Na9WstxUKKsTZK4R2b1pgpLkdFhwKj7KsdfNrYG6EhuB8W6x7qjlD1Vim8XZWqx9SOzstx-j7Mquhmo_tpIrXqM761LLI8XEGoo_enq-Mu3dqhHdqEdoLU5UtPXZFYktEUsf3_5Z4XLqXZFgklIvHjbovUnri8EW3eSMGvIMAEx9zN3T-XeuugkozNeVv4mhPLWuELNeoryE-TMHzLp-9h9moolISkf.U1Yk0ZDqpy4vpgwzUvI-U6KY5y-8Ih-YThqMuylk_UlG0A-V5HczPfKM5gK1ThI8mhC0Iybqmh7GuZN_UfKspyfqnfKWpyfqn16d0AdY5HDsPjwxnH0kPdtknjD4g1nvnjD0pvbqn0KzIjY3nWf0uy-b5HDYPWIxnWDknHb0mhbqnW0Yg1DdPfKVm1YkPHbdrHRzrjmvg1RLnHb1njf1ndtsg100TgKGujYs0Z7Wpyfqn0KzuLw9u1Ys0AqvUjYknjcknBdbX-tknjc4nadbX-tknjfLPiYkg1Dsrj0YQHKxnH03rHmVuZGxnH04nWcVnNtknjb4PBdbX-tknH0znBdbX-tknH01nBdbX-tknH0vPBdbX-tknH0vridbX-tknHDvnadbX-tknHfsPaYkg1DkPjczQywlg1DkPjn4QHFxnHDdPWfVuZGxnHDdP1cVuZGxnHDvnjmVnNtknHmLriYvg1DznadbX-tknWDLniYkg1DznW6YQywlg1Dzn1cYQywlg1Dzn1fLQH7xnHcYP10VnNtknWRvnzdbX-tknWRLPidbX-tknWmkriYzg1DzPWnsQH7xnHcvPW0VnNtknWTkPzdbX-tknWT1PzYkg1DzP1TYQywlg1csPYsVPdtznjInyaYLg1b4riYL0A7B5HKxn0K-ThTqn0KsTjY3PWDsnjbdrHD0UMus5H08nj0snj0snj00Ugws5H00uAwETjYs0ZFJ5H00uANv5gKW0AuY5H00TA6qn0KET1Ys0AFL5HDs0A4Y5H00TLCq0ZwdT1Y4n1DLrHfsP161rj63PjR3nWTY0ZF-TgfqnHf4PW6znjfkPHb3P0K1pyfquHD4nHcvmWfsnj0snyuWnfKWTvYqnRDYPWIanjm1fHNjPjczr0K9m1Yk0ZK85H00TydY5H00Tyd15H00XMfqn0KVmdqhThqV5HDsPjuxn7tsg100uA78IyF-gLK_my4GuZnqn7tsg1Kxn0Ksmgwxuhk9u1Ys0AwYpyfqn0K-IA-b5iYk0A71TAPW5H00IgKGUhPW5H00Tydh5HDv0AuWIgfqn0KhXh6qn0Khmgfqn0KlTAkdT1Ys0A7buhk9u1Yk0Akhm1Ys0APzm1Ykrjcs&ck=1592.9.152.167.178.135.202.2057&shh=www.baidu.com&sht=57095150_1_oem_dg&wd=Invtrigen&issp=1&f=3&ie=utf-8&rqlang=cn&tn=57095150_1_oem_dg&oq=pY&gt;S2.0/&lt;T&prefixsug=Invtrigen&rsp=0&bc=110101&us=1.113282.3.0.6.3050.0.0) |
| pWYE3223 | pESC-LEU | Addgene（Plasmid #20120） |
| pWYE3224 | pWYE3222 derivative, *P_ScGAL1_*-*Cas9* | This study |
| pWYE3225 | pWYE3223 derivative, *P_ScSNR52_-ScrDNA*gRNA | This study |
| pWYE3226 | pWYE3200 derivative, Zeo^R^,  *ScrDNA*upHA-*P_ScTEF1_-gfpmut3a-Sc*rDNAdownHA | This study |
| pWYE3227 | pWYE3200 derivative, Zeo^R^ ,  *ScScALG9*partial-*gfpmut3a*partial-*OpMOX*partial | This study |
| pWYE3228 | pMD19 T derivative, Amp^R^,  OP*OpMOX*partial-*HSA*partial-*cadA*partial-*TAL* partial | This study |
| pWYE3229 | pWYE3201 derivative,  G418^R^, *OpADE2*upHA-*P_ScSNR52_-OpURA3*gRNA*-*OpADE2*downHA | This study |
| pWYE3230 | pWYE3200 derivative,  Zeo^R^, rDNAupHA- *P_ScTEF1_*-*TAL-P_ScTPI1_-4CL-P_ScTEF2_-STS -*rDNAdownHA | This study |
| pWYE3231 | pWYE3200 derivative,  Zeo^R^, rDNAupHA-*P_ScTEF1_-cadA-*rDNAdownHA | This study |
| pWYE3232 | pWYE3200 derivative,  Zeo^R^, rDNAupHA-*P_ScTEF1_-HSA-*rDNAdownHA | This study |
| pWYE3233 | pWYE3201 derivative,  G418^R^, *OpURA3*upHA-*P_ScSNR52_-OpADE2*gRNA-*Op URA3*downHA | This study |

^a^ Bacillus Genetic Stock Center; ^b^ The zeocin resistance gene *zeo^R^* was replaced by the G418 resistance gene *G418^R^*.

**Table S2. Primers used in this study**

| **Primer** | | **Sequence(5'-to-3')** | | | **Description/Source** | |
| --- | --- | --- | --- | --- | --- | --- |
| Primers for construction of pWYE3208 | | | | |  | |
| P1 | | GATTTTGGTCATGCATGAGATCAGATCTTAGAGTGTATAACAAAGGAT | | | *OpMET2* downstream  forward | |
| P2 | | CCCCTGGAGCACTAGTTTTAGCATCTGCCAGATTGAGG | | | *OpMET2* downstream  reverse | |
| P3 | | AGATGCTAAAACTAGTGCTCCAGGGGCACTCAGCTTAG | | | *OpMET2* upstream  forward | |
| P4 | | GAAGCTATGCGTCGGTGTGCGAGTTGAACTCTG | | | *OpMET2* upstream  reverse | |
| P5 | | GCACACCGACGCATAGCTTCAAAATGTTTCTAC | | | *Cas9* expression cassette forward | |
| P6 | | CTCTTCTGAGATGAGTTTTTGTTCTAGAATAAATCGTAAAGACATAAGAG | | | *Cas9* expression cassette reverse | |
| Primers for detection of pWYE3208 integration | | | | |  | |
| P7 | | GAGTTGTCCAGCAGGAGCCCATG | | | forward | |
| P8 | | GCTATTTGTGCCGATATCTAAGCC | | | reverse | |
| Primers for detection of pWYEN integration | | | | | | |
| P9 | | CTCTCATCAGCAGCAGCCGTCCG | | | forward | |
| P10 | | TATGAGTGAAAGCATAATCATAC | | | reverse | |
| Primers for construction of pWYE3209 | | | | | | |
| P11 | | ATTTTGGTCATGCATGAGATCAGATCTATAGAGGTTAAATTAATTCAATTAC | | | *OpADE2* downstream  forward | |
| P12 | | GGTACAACGGGCATGCACTAGTGGTACCAAGCAGGACTTTCAAATCTTC | | | *OpADE2* downstream  reverse | |
| P13 | | GGTACCACTAGTGCATGCCCGTTGTACCTCGTTCGCCAG | | | *OpADE2* upstream  forward | |
| P14 | | CATACATTATCTTTTCAAAGAGTAAATTAAATTAAATTAATATATG | | | *OpADE2* upstream  reverse | |
| P15 | | AATTTAATTTACTCTTTGAAAAGATAATGTATG | | | *P_ScSNR52_* forward | |
| P16 | | CTCTAAAACTGAAATCAGAAATCGTCAAGATCATTTATCTTTCACTGC | | | *P_ScSNR52_* reverse | |
| P17 | | ATAAATGATCTTGACGATTTCTGATTTCAGGTTTTAGAGCTAGAAATAGC | | | crRNA forward | |
| P18 | | CATTTTGAAGCTATGGTGTGTGGGGGATCCAGACATAAAAAACAAAAAAAGCACC | | | crRNA reverse | |
| Primers for detection of *OpLEU2* deletion | | | | | | |
| P19 | | GTGCTCCTCAAAGCTGACCGTCTA | | | forward | |
| P20 | | CCGCAAACCTCCCTGTCGGGCACT | | | reverse | |
| Primers for construction of pWYE3210 | | | | | | |
| P21 | | GATTTTGGTCATGCATGAGATCAGATCTAGTTTGCCAAGTATGCCAG | | | *OpLEU2* upstream  forward | |
| P22 | | AGTTGGGTGGTCGCTTTCTGATGATTGCAAAATGATGCAAC | | | *OpLEU2* upstream  reverse | |
| P23 | | ATCAGAAAGCGACCACCCAACT | | | *gfpmut3a* expression cassette  forward | |
| P24 | | GGATCCGCACAAACGAAGGTC | | | *gfpmut3a* expression cassette  reverse | |
| P25 | | AAGTGAGACCTTCGTTTGTGCGGATCCGTAGGATCTCGAATAATTCC | | | *OpLEU2* downstream  forward | |
| P26 | | TTGAAGCTATGGTGTGTGGGGGATCCTCTCTTTTGATGGCATTGAAG | | | *OpLEU2* downstream  reverse | |
| Primers for construction of pWYE3211 | | | | | | |
| P11 | | ATTTTGGTCATGCATGAGATCAGATCTATAGAGGTTAAATTAATTCAATTAC | | | *OpADE2* downstream  forward | |
| P12 | | GGTACAACGGGCATGCACTAGTGGTACCAAGCAGGACTTTCAAATCTTC | | | *OpADE2* downstream  reverse | |
| P13 | | GGTACCACTAGTGCATGCCCGTTGTACCTCGTTCGCCAG | | | *OpADE2* upstream  forward | |
| P14 | | CATACATTATCTTTTCAAAGAGTAAATTAAATTAAATTAATATATG | | | *OpADE2* upstream  reverse | |
| P15 | | AATTTAATTTACTCTTTGAAAAGATAATGTATG | | | *P_ScSNR52_* forward | |
| P27 | | CTCTAAAACATCTAAGGTCGCCAGCAGACGATCATTTATCTTTCACTGC | | | *P_ScSNR52_* reverse | |
| P28 | | ATAAATGATCGTCTGCTGGCGACCTTAGATGTTTTAGAGCTAGAAATAGC | | | crRNA forward | |
| P18 | | CATTTTGAAGCTATGGTGTGTGGGGGATCCAGACATAAAAAACAAAAAAAGCACC | | | crRNA reverse | |
| Primers for detection of  *OpURA3* deletion | | | | | | |
| P29 | | ACTTGAGTCAGACGAGGGTAAGG | | | forward | |
| P30 | | AGCGAGCGAAAACGGCCGATTGG | | | reverse | |
| Primers for restoration of *OpADE2* | | | | | | |
| P31 | | CTCTCATCAGCAGCAGCCGTCCG | | | forward | |
| P32 | | CTGCTTGGCCGGTGAATCTGCACC | | | reverse | |
| Primers for restoration of *OpMET2* | | | | | | |
| P33 | | CTTCATCAACAACTTCCCAGAC | | | forward | |
| P34 | | GCGAAGGTTCGAGCGATGAGAG | | | reverse | |
| Primers for construction of pWYE3212 | | | | | | |
| P35 | | GATTTTGGTCATGCATGAGATCAGATCTAAAACAGAAGAGACAGAATGG | | | *OpURA3* upstream  forward | |
| P36 | | AGTTGGGTGGTCGCTTTCTGATGTTGATTATTATTCAGGGAAATG | | | *OpURA3* upstream  reverse | |
| P37 | | ATCAGAAAGCGACCACCCAACT | | | *gfpmut3a* expression cassette  forward | |
| P38 | | GGATCCGCACAAACGAAGGTC | | | *gfpmut3a* expression cassette  reverse | |
| P39 | | AAGTGAGACCTTCGTTTGTGCGGATCCCGGCTTTCAGTTCTATATAC | | | *OpURA3* downstream  forward | |
| P40 | | TTGAAGCTATGGTGTGTGGGGGATCCGTTCTTGCCGTGTCTTCTAAG | | | *OpURA3* downstream  reverse | |
| Primers for construction of pWYE3213 | | | | | | |
| P11 | | ATTTTGGTCATGCATGAGATCAGATCTATAGAGGTTAAATTAATTCAATTAC | | | *OpADE2* downstream  forward | |
| P12 | | GGTACAACGGGCATGCACTAGTGGTACCAAGCAGGACTTTCAAATCTTC | | | *OpADE2* downstream  reverse | |
| P13 | | GGTACCACTAGTGCATGCCCGTTGTACCTCGTTCGCCAG | | | *OpADE2* upstream  forward | |
| P14 | | CATACATTATCTTTTCAAAGAGTAAATTAAATTAAATTAATATATG | | | *OpADE2* upstream  reverse | |
| P15 | | AATTTAATTTACTCTTTGAAAAGATAATGTATG | | | *P_ScSNR52_* forward | |
| P41 | | CTCTAAAACCCATCCAGACTTAGAACAACGATCATTTATCTTTCACTGC | | | *P_ScSNR52_* reverse | |
| P42 | | ATAAATGATCGTTGTTCTAAGTCTGGATGGGTTTTAGAGCTAGAAATAGC | | | crRNA forward | |
| P18 | | CATTTTGAAGCTATGGTGTGTGGGGGATCCAGACATAAAAAACAAAAAAAGCACC | | | crRNA reverse | |
| Primers for construction of pWYE3214 | | | | | | |
| P43 | | GATTTTGGTCATGCATGAGATCAGATCTTTCCGTACAACGAAATGGTTG | | | *OpHIS3* upstream  forward | |
| P44 | | AGTTGGGTGGTCGCTTTCTGATTTCAGTTTTATTGTAATTTAC | | | *OpHIS3* upstream  reverse | |
| P45 | | ATCAGAAAGCGACCACCCAACT | | | *gfpmut3a* expression cassette  forward | |
| P46 | | GGATCCGCACAAACGAAGGTC | | | *gfpmut3a* expression cassette  reverse | |
| P47 | | AAGTGAGACCTTCGTTTGTGCGGATCCTAGACCGGTGCGGGGTGTGC | | | *OpHIS3* downstream  forward | |
| P48 | | TTGAAGCTATGGTGTGTGGGGGATCCCGATTGGTCCAATCGAACAGG | | | *OpHIS3* downstream  reverse | |
| Primers for construction of pWYE3215 | | | | | | |
| P49 | | GGTGCTTTTTTTGTTTTTTATGTCTGGATCCTCTTTGAAAAGATAATGTATGA | | | *OpHIS3*-gRNA expression cassette  forward | |
| P50 | | CCCAACAGTTGCGCAGCCTGAGACATAAAAAACAAAAAAAGCACC | | | *OpHIS3*-gRNA expression cassette  reverse | |
| P51 | | CAGGCTGCGCAACTGTTGGGTCTTTGAAAAGATAATGTATGA | | | *OpLEU2*-gRNA expression cassette  forward | |
| P52 | | CATTTTGAAGCTATGGTGTGTGGGGAGACATAAAAAACAAAAAAAGCACC | | | *OpLEU2*-gRNA expression cassette  reverse | |
| Primers for detection of integration at *OpURA3* | | | | | | |
| P53 | | ACTTGAGTCAGACGAGGGTAAGG | | | forward | |
| P54 | | AGCGAGCGAAAACGGCCGATTGG | | | reverse | |
| Primers for detection of integration at *OpLEU2* | | | | | | |
| P55 | | GTGCTCCTCAAAGCTGACCGTCTA | | | forward | |
| P56 | | CCGCAAACCTCCCTGTCGGGCACT | | | reverse | |
| Primers for detection of integration at *OpHIS3* | | | | | | |
| P57 | | GCAAGTATTCCTGCTACCGACTTG | | | forward | |
| P58 | | CTTCAGCTCTGTAGAGTACTGCAG | | | reverse | |
| Primers for construction of pWYE3216 | | | | | | |
| P59 | | GATTTTGGTCATGCATGAGATCAGATCTTGAGCTTGAGAAACGCCAGTC | | | *OpLEU2* upstream  forward | |
| P60 | | GCTAAAAAAACTCTACATAACAAAGTGATTGCAAAATGATGCAACTA | | | *OpLEU2* upstream  reverse | |
| P61 | | ACTTTGTTATGTAGAGTTTTTTTAGC | | | *STS* expression  cassette forward | |
| P62 | | TTTGAAAGATGATACTCTTTATTTCTAG | | | *STS* expression  cassette reverse | |
| P63 | | AATAAAGAGTATCATCTTTCAAAGTAGGATCTCGAATAATTCC | | | *OpLEU2* downstream  forward | |
| P64 | | TTGAAGCTATGGTGTGTGGGGGATCCATGCGCGCTTTTCGCTGAGGT | | | *OpLEU2* downstream  reverse | |
| Primers for construction of pWYE3217 | | | | | | |
| P65 | | GATTTTGGTCATGCATGAGATCAGATCTGAGGGCGTGACGCATAATGACG | | | *OpURA3* upstream  forward | |
| P66 | | CAAAGTTGGGTGGTCGCTTTCTGGTTGATTATTATTCAGGGAAATG | | | *OpURA3* upstream  reverse | |
| P67 | | CAGAAAGCGACCACCCAACTTTG | | | *TAL* expression  cassette forward | |
| P68 | | TTTGAAAGATGATACTCTTTATTTC | | | *TAL* expression  cassette reverse | |
| P69 | | TAAAGAGTATCATCTTTCAAACGGCTTTCAGTTCTATATAC | | | *OpURA3* downstream  forward | |
| P70 | | TTGAAGCTATGGTGTGTGGGGGATCCACAGGTTGTGTCTGCCTCTTC | | | *OpURA3* downstream  reverse | |
| Primers for construction of pWYE3218 | | | | | | |
| P71 | | GATTTTGGTCATGCATGAGATCAGATCTCCAAAGGCCACGGTTCAGCAG | | | *OpHIS3* upstream  forward | |
| P72 | | GTTCCTAGATATAATCTCGAAGGTTCAGTTTTATTGTAATTTAC | | | *OpHIS3* upstream  reverse | |
| P73 | | CCTTCGAGATTATATCTAGGAAC | | | *4CL* expression  cassette forward | |
| P74 | | TTTGAAAGATGATACTCTTTATTCC | | | *4CL* expression  cassette reverse | |
| P75 | | TAAAGAGTATCATCTTTCAAATAGACCGGTGCGGGGTGTGC | | | *OpHIS3* downstream  forward | |
| P76 | | TTGAAGCTATGGTGTGTGGGGGATCCCTGTTTAACTTGGTAGTTGATC | | | *OpHIS3* downstream  reverse | |
| Primers for construction of pWYE3219 | | | | | | |
| P1 | | GATTTTGGTCATGCATGAGATCAGATCTTAGAGTGTATAACAAAGGAT | | | *OpMET2* downstream  forward | |
| P2 | | CCCCTGGAGCACTAGTTTTAGCATCTGCCAGATTGAGG | | | *OpMET2* downstream  reverse | |
| P3 | | AGATGCTAAAACTAGTGCTCCAGGGGCACTCAGCTTAG | | | *OpMET2* upstream  forward | |
| P77 | | GATCGTTCTCCGCGTCGAGTCGGTGTGCGAGTTGAACTCTG | | | *OpMET2* upstream  reverse | |
| P78 | | CACACCGACTCGACGCGGAGAACGATCTCC | | | *P_OpMOX_* forward | |
| P79 | | TATAATCCATGTGTGTTGTACTTTAGATTGATG | | | *P_OpMOX_* reverse | |
| P80 | | CAATCTAAAGTACAACACACATGGATTATAAAGATGACGATG | | | *Cas9* forward | |
| P6 | | CTCTTCTGAGATGAGTTTTTGTTCTAGAATAAATCGTAAAGACATAAGAG | | | *Cas9* reverse | |
| Primers for detection of pWYE3219 integration | | | | | | |
| P81 | | GAGTTGTCCAGCAGGAGCCCATG | | | forward | |
| P82 | | GCTCGCCAGCCACCGTGGTCCGC | | | reverse | |
| Primers for construction of pWYE3220 | | | | | | |
| P11 | | ATTTTGGTCATGCATGAGATCAGATCTATAGAGGTTAAATTAATTCAATTAC | | | *OpADE2* downstream  forward | |
| P12 | | GGTACAACGGGCATGCACTAGTGGTACCAAGCAGGACTTTCAAATCTTC | | | *OpADE2* downstream  reverse | |
| P13 | | GGTACCACTAGTGCATGCCCGTTGTACCTCGTTCGCCAG | | | *OpADE2* upstream  forward | |
| P14 | | CATACATTATCTTTTCAAAGAGTAAATTAAATTAAATTAATATATG | | | *OpADE2* upstream  reverse | |
| P15 | | AATTTAATTTACTCTTTGAAAAGATAATGTATG | | | *P_ScSNR52_* forward | |
| P83 | | CTCTAAAACTTGTCTATCCAAACGTCTATGATCATTTATCTTTCACTGC | | | *P_ScSNR52_* reverse | |
| P84 | | ATAAATGATCATAGACGTTTGGATAGACAAGTTTTAGAGCTAGAAATAGC | | | crRNA forward | |
| P18 | | CATTTTGAAGCTATGGTGTGTGGGGGATCCAGACATAAAAAACAAAAAAAGCACC | | | crRNA reverse | |
| Primers for construction of pWYE3221 | | | | | | |
| P85 | | GATTTTGGTCATGCATGAGATCAGATCTTTGCCATAGGCTAGTAATC | | | rDNA upstream  forward | |
| P86 | | AGTTGGGTGGTCGCTTTCTGATTGATCGGACGGGAAACGGTGC | | | rDNA upstream  reverse | |
| P87 | | ATCAGAAAGCGACCACCCAACT | | | *gfpmut3a* expression cassette  forward | |
| P88 | | GGATCCGCACAAACGAAGGTC | | | *gfpmut3a* expression cassette  reverse | |
| P89 | | AAGTGAGACCTTCGTTTGTGCGGATCCCCAGCGCCAGATAACAAACAG | | | rDNA downstream  forward | |
| P90 | | TTGAAGCTATGGTGTGTGGGGGATCCGGGTTTAGACCGTCGTGAGACAG | | | rDNA downstream  reverse | |
| Primers for detection of *gfpmut3a* integration at rDNA sites in *O. polymorpha* | | | | | | |
| P91 | | CTAACTGCATCCATATAGCCCTC | | | forward | |
| P92 | | TGTGCCCATTAACATCACCATC | | | reverse | |
| Primers for detection of *cadA* integration at rDNA sites | | | | | | |
| P93 | | CTAACTGCATCCATATAGCCCTC | | | forward | |
| P94 | | GTCGTTCGGGTAAACAATCTGGAAG | | | reverse | |
| Primers for detection of *HSA* integration at rDNA sites | | | | | | |
| P95 | | CTAACTGCATCCATATAGCCCTC | | | forward | |
| P96 | | ACCCAAGTCCTTGAATCTGTGAGC | | | reverse | |
| Primers for detection of *TAL-4CL-STS* integration at rDNA sites | | | | | | |
| P97 | | CTAACTGCATCCATATAGCCCTC | | | forward | |
| P98 | | GTAGGCGCAAGAAGCTTCAATTTG | | | reverse | |
| Primers for construction of pWYE3224 | | | | | | |
| P99 | | TTAAGCTTGGTACCGAGCTCGGATCCATGGATTATAAAGATGACGATG | | | *Cas9* forward | |
| P100 | | CACTGTGCTGGATATCTGCAGAATTCATAAATCGTAAAGACATAAGAG | | | *Cas9* reverse | |
| Primers for construction of pWYE3225 | | | | | | |
| P101 | | GAAGTTGATTTCCGAAGAAGACCTCGAGTCTTTGAAAAGATAATGTATG | | | *P_ScSNR52_* forward | |
| P102 | | GCTCTAAAACCATCGTATATTATAATAGATGATCATTTATCTTTCACTGC | | | *P_ScSNR52_* reverse | |
| P103 | | ATAAATGATCATCTATTATAATATACGATGGTTTTAGAGCTAGAAATAGC | | | crRNA forward | |
| P104 | | AGAGCGGATCTTAGCTAGCCGCGGTACCAGACATAAAAAACAAAAAAAGCACCACCGACTCGGTGC | | | crRNA reverse | |
| Primers for construction of pWYE3226 | | | | | | |
| P105 | | GATTTTGGTCATGCATGAGATCAGATCTACCTACCGACCAACTTTCATG | | | *ScrDNA* upstream  forward | |
| P106 | | AGTTGGGTGGTCGCTTTCTGATAGGACATGCCTTTGATATGA | | | *ScrDNA* upstream  reverse | |
| P107 | | ATCAGAAAGCGACCACCCAACT | | | *gfpmut3a* expression cassette  forward | |
| P108 | | GGATCCGCACAAACGAAGGTC | | | *gfpmut3a* expression cassette  reverse | |
| P109 | | AAGTGAGACCTTCGTTTGTGCGGATCCACAAATCAGACAACAAAGGCT | | | *ScrDNA* downstream  forward | |
| P110 | | TTGAAGCTATGGTGTGTGGGGGATCCGCGAAACCACAGCCAAGGGAAC | | | *ScrDNA* downstream  reverse | |
| Primers for detection of *gfpmut3a* integration at rDNA sites in *S. cerevisiae* | | | | | | |
| P111 | | ATCTCTTCCCGTCATTATCGCC | | | forward | |
| P112 | | TGTGCCCATTAACATCACCATC | | | reverse | |
| Primers for construction of pWYE3227 | | | | | | |
| P113 | | GATTTTGGTCATGCATGAGATCAGATCTGAGATTATGGCCATTATGGGCAT | | | *SC**ScALG9* forward | |
| P114 | | CTTCAGCCAGTGCTCTGCACATCAATGTAACGAACACCGTAC | | | *SCScALG9* reverse | |
| P115 | | GTGCAGAGCACTGGCTGAAGTG | | | *OpMOX* forward | |
| P116 | | CTTCTGAGCAACCGGGTCACC | | | *OpMOX* reverse | |
| P117 | | GTGACCCGGTTGCTCAGAAGATGAGTAAAGGAGAAGAAC | | | *gfpmut3a* forward | |
| P118 | | TTGAAGCTATGGTGTGTGGGGGATCCTTATTTGTATAGTTCATCCATG | | | *gfpmut3a* reverse | |
| Primers for construction of pWYE3228 | | | | | | |
| P119 | | GTACCCGGGGATCCTCTAGAGATGTGCAGAGCACTGGCTGAAG | | | *OpMOX* forward | |
| P120 | | GATGAAAGTAACCCACTTCATCTTCTGGGCAACTGGGTCAC | | | *OpMOX* reverse | |
| P121 | | ATGAAGTGGGTTACTTTCATC | | | *HSA* forward | |
| P122 | | ATTCAGCCTTTGGGAATCTTTG | | | *HSA* reverse | |
| P123 | | AAGATTCCCAAAGGCTGAATTTGGAATTATGTGAAGAAATTTC | | | *cadA* forward | |
| P124 | | CGATAGTTGCATGTTGAAATT | | | *cadA* reverse | |
| P125 | | AATTTCAACATGCAACTATCGATGGACCAATTGAGATATTACATG | | | *TAL* forward | |
| P126 | | TTGCATGCCTGCAGGTCGACGATTCATCTGAACAAGATGATGGA | | | *TAL* reverse | |
| Primers for construction of pWYE3229 | | | | | | |
| P11 | ATTTTGGTCATGCATGAGATCAGATCTATAGAGGTTAAATTAATTCAATTAC | | | *OpADE2* downstream  forward | | |
| P12 | GGTACAACGGGCATGCACTAGTGGTACCAAGCAGGACTTTCAAATCTTC | | | *OpADE2* downstream  reverse | | |
| P13 | GGTACCACTAGTGCATGCCCGTTGTACCTCGTTCGCCAG | | | *OpADE2* upstream  forward | | |
| P14 | CATACATTATCTTTTCAAAGAGTAAATTAAATTAAATTAATATATG | | | *OpADE2* upstream  reverse | | |
| P15 | AATTTAATTTACTCTTTGAAAAGATAATGTATG | | | *P_ScSNR52_* forward | | |
| P127 | CTCTAAAACTCAAATTAAGTAGTCTGCTGGATCATTTATCTTTCACTGC | | | *P_ScSNR52_* reverse | | |
| P128 | ATAAATGATCCAGCAGACTACTTAATTTGAGTTTTAGAGCTAGAAATAGC | | | crRNA forward | | |
| P18 | CATTTTGAAGCTATGGTGTGTGGGGGATCCAGACATAAAAAACAAAAAAAGCACC | | | crRNA reverse | | |
| Primers for construction of pWYE3230 | | | | | | |
| P85 | GATTTTGGTCATGCATGAGATCAGATCTTTGCCATAGGCTAGTAATC | | | rDNA upstream  forward | | |
| P86 | AGTTGGGTGGTCGCTTTCTGATTGATCGGACGGGAAACGGTGC | | | rDNA upstream  reverse | | |
| P129 | ATCAGAAAGCGACCACCCAACT | | | *P_ScTEF1_*-*TAL expression cassette*  forward | | |
| P130 | CTGATGGGTTCCTAGATATAATCTCGAAGGTTTGAAAGATGATACTCTTTATTTC | | | *P_ScTEF1_*-*TAL expression cassette*  reverse | | |
| P131 | CCTTCGAGATTATATCTAGGAACCCATCAG | | | *P_ScTPI1_*-*4CL expression cassette*  forward | | |
| P132 | TTTGAAAGATGATACTCTTTATTCCTACATAAGTAAATGAGTTTATATATTACAAACCGTTAGCCAACTTAG | | | *P_ScTPI1_*-*4CL expression cassette*  reverse | | |
| P133 | AGGAATAAAGAGTATCATCTTTCAAAACTTTGTTATGTAGAGTTTTTTTAG | | | *P_ScTEF2_*-*STS expression cassette*  forward | | |
| P134 | CCTCTTCTGAGATGAGTTTTTGTTCTAGACTGTGCTGGATATCTGCAGAATTC | | | *P_ScTEF2_*-*STS expression cassette*  reverse | | |
| P135 | TAAAGAGTATCATCTTTCAAACCAGCGCCAGATAACAAACAG | | | rDNA downstream  forward | | |
| P90 | TTGAAGCTATGGTGTGTGGGGGATCCGGGTTTAGACCGTCGTGAGACAG | | | rDNA downstream  reverse | | |
| Primers for construction of pWYE3231 | | | | | | |
| P85 | GATTTTGGTCATGCATGAGATCAGATCTTTGCCATAGGCTAGTAATC | | rDNA upstream  forward | | |  |
| P86 | AGTTGGGTGGTCGCTTTCTGATTGATCGGACGGGAAACGGTGC | | rDNA upstream  reverse | | |  |
| P129 | ATCAGAAAGCGACCACCCAACT | | *P_ScTEF1_* forward | | |  |
| P136 | TTTGTAATTAAAACTTAGATTAG | | *P_ScTEF1_* reverse | | |  |
| P137 | CTAATCTAAGTTTTAATTACAAAATGAATGTTATTGCTATCTTG | | *cadA* forward | | |  |
| P138 | TTTGAAAGATGATACTCTTTATTTCTAGACAGTTATATA TTATTTCTTAGATTCTTCTT | | *cadA* reverse | | |  |
| P135 | TAAAGAGTATCATCTTTCAAACCAGCGCCAGATAACAAACAG | | rDNA downstream  forward | | |  |
| P90 | TTGAAGCTATGGTGTGTGGGGGATCCGGGTTTAGACCGTCGTGAGACAG | | rDNA downstream  reverse | | |  |
| Primers for construction of pWYE3232 | | | | | | |
| P85 | GATTTTGGTCATGCATGAGATCAGATCTTTGCCATAGGCTAGTAATC | | | rDNA upstream  forward | | |
| P86 | AGTTGGGTGGTCGCTTTCTGATTGATCGGACGGGAAACGGTGC | | | rDNA upstream  reverse | | |
| P129 | ATCAGAAAGCGACCACCCAACT | | | *P_ScTEF1_* forward | | |
| P136 | TTTGTAATTAAAACTTAGATTAG | | | *P_ScTEF1_* reverse | | |
| P139 | CTAATCTAAGTTTTAATTACAAA ATGAAGTGGGTTACTTTCATC | | | *HSA* forward | | |
| P140 | TTTGAAAGATGATACTCTTTATTTCTAGACAGTTATATATTACAAACCCAAAGCAGCT | | | *HSA* reverse | | |
| P135 | TAAAGAGTATCATCTTTCAAACCAGCGCCAGATAACAAACAG | | | rDNA downstream  forward | | |
| P90 | TTGAAGCTATGGTGTGTGGGGGATCCGGGTTTAGACCGTCGTGAGACAG | | | rDNA downstream  reverse | | |
| Primers for qPCR | | | | | | |
| P141 | TCACGGATAGTGGCTTTGG | | | *ScALG9* forward | | |
| P142 | AGTGATACCATTCACGTCCC | | | *ScALG9* reverse | | |
| P143 | GGTGAAATGGCTGACTGT | | | *HSA* forward | | |
| P144 | TTGTCGTGGAAAGCAGTA | | | *HSA* reverse | | |
| P145 | TGGTCCAAACACTATGAAG | | | *cadA* forward | | |
| P146 | TAGCGATGTATTGTTCTGC | | | *cadA* reverse | | |
| P147 | ACTTTCGGGTATGGTGTTCA | | | *gfpmut3a* forward | | |
| P148 | TGTAGTTCCCGTCATCTTTG | | | *gfpmut3a* reverse | | |
| P149 | TTCCTCATCACCTCCACCAAG | | | *OpMOX* forward | | |
| P150 | TCCGCAAGAAATCACAATCTG | | | *OpMOX* reverse | | |
| P151 | TCGTCAAACATCCACCATCTCC | | | *TAL* forward | | |
| P152 | TCTCAAACCGGACAAAGTTTG | | | *TAL* reverse | | |
| Primers for deletion of *OpLEU2* by CRISPR-Cas9 system | | | | | | |
| P153 | AAGGTGGAGATGGTGTACTG | | | *OpLEU2* upstream  forward | | |
| P154 | GAGATCCTACGATTGCAAAATGATGCAAC | | | *OpLEU2* upstream  reverse | | |
| P155 | CATTTTGCAATCGTAGGATCTCGAATAATTCC | | | *OpLEU2* downstream  forward | | |
| P156 | TTCGCTGAGGTTGTCTCTGTC | | | *OpLEU2* downstream  reverse | | |
| Primers for deletion of *OpURA3* by CRISPR-Cas9 system | | | | | | |
| P157 | GAGGGCGTGACGCATAATGACG | | | *OpURA3* upstream  forward | | |
| P158 | TAGAACTGAAAGCCG GTTGATTATTATTCAGGGAAATG | | | *OpURA3* upstream  reverse | | |
| P159 | TGAATAATAATCAAC CGGCTTTCAGTTCTATATACATC | | | *OpURA3* downstream  forward | | |
| P160 | GCAAAGCTATTTAGGCCGTCTCG | | | *OpURA3* downstream  reverse | | |
| Primers for point mutation of *OpURA3* by CRISPR-Cas9 system | | | | | | |
| P161 | GAGGGCGTGACGCATAATGACG | | | *OpURA3* upstream  forward | | |
| P162 | TGTTTGCTTGGATT**A**CATCAAATTAAGTAGTCTGC | | | *OpURA3* upstream  reverse | | |
| P163 | TACTTAATTTGATG**T**AATCCAAGCAAACAAACCTCTG | | | *OpURA3* downstream  forward | | |
| P164 | GCTGGAGCTTCCGCCGCAACTAC | | | *OpURA3* downstream  reverse | | |
| Primers for replacemet of *OpADE2* by the fragment *OpADE2*UHA-*G418^R^* expression cassette-*OpADE2*DHA | | | | | | |
| P165 | CCGTTGTACCTCGTTCGCCAGCC | | | *OpADE2* upstream  forward | | |
| P166 | CATTTTGAAGCTATGGTGTGTGGG GCTGACTTGGATATTATTATC | | | *OpADE2* upstream  reverse | | |
| P167 | CCCACACACCATAGCTTCAAAATG | | | *G418^R^* expression cassette forward | | |
| P168 | AGCTTGCAAATTAAAGCCTTCGAGC | | | *G418^R^* expression cassette reverse | | |
| P169 | GCTCGAAGGCTTTAATTTGCAAGCTATAGAGGTTAAATTAATTCAATTAC | | | *OpADE2* upstream  forward | | |
| P170 | AAGCAGGACTTTCAAATCTTCC | | | *OpADE2* upstream  reverse | | |
| Primers for replacemet of *OpLEU2* by the fragment *OpLEU2*UHA-*G418^R^* expression cassette-*OpLEU2*DHA | | | | | | |
| P171 | CGCCAGTCTAGAAACAAGGTGGAG | | | *OpLEU2* upstream  forward | | |
| P172 | CATTTTGAAGCTATGGTGTGTGGG GATTGCAAAATGATGCAACTAT | | | *OpLEU2* upstream  reverse | | |
| P167 | CCCACACACCATAGCTTCAAAATG | | | *G418^R^* expression cassette forward | | |
| P168 | AGCTTGCAAATTAAAGCCTTCGAGC | | | *G418^R^* expression cassette reverse | | |
| P173 | GCTCGAAGGCTTTAATTTGCAAGCTGTAGGATCTCGAATAATTCCTA | | | *OpLEU2* upstream  forward | | |
| P174 | GGCTCCGATTCCTGCTGCCGCAC | | | *OpLEU2* upstream  reverse | | |
| Primers for replacemet of *OpURA3* by the fragment *OpURA3*UHA-*G418^R^* expression cassette-*OpURA3*DHA | | | | | | |
| P175 | GAGGGCGTGACGCATAATGACG | | | *OpURA3* upstream  forward | | |
| P176 | CATTTTGAAGCTATGGTGTGTGGG GTTGATTATTATTCAGGGAAATG | | | *OpURA3* upstream  reverse | | |
| P167 | CCCACACACCATAGCTTCAAAATG | | | *G418^R^* expression cassette forward | | |
| P168 | AGCTTGCAAATTAAAGCCTTCGAGC | | | *G418^R^* expression cassette reverse | | |
| P177 | GCTCGAAGGCTTTAATTTGCAAGCTCGGCTTTCAGTTCTATATACATC | | | *OpURA3* upstream  forward | | |
| P178 | GCAAAGCTATTTAGGCCGTCTCG | | | *OpURA3* upstream  reverse | | |
| Primers for replacemet of *OpHIS3* by the fragment *OpHIS3*UHA-*G418^R^* expression cassette-*OpHIS3*DHA | | | | | | |
| P179 | GACCGTTCACCAGGCACTGATTC | | | *OpHIS3* upstream  forward | | |
| P180 | CATTTTGAAGCTATGGTGTGTGGG ATTTATGCTGAGATTAGTCAGAC | | | *OpHIS3* upstream  reverse | | |
| P167 | CCCACACACCATAGCTTCAAAATG | | | *G418^R^* expression cassette forward | | |
| P168 | AGCTTGCAAATTAAAGCCTTCGAGC | | | *G418^R^* expression cassette reverse | | |
| P181 | GCTCGAAGGCTTTAATTTGCAAGCTTAGACCGGTGCGGGGTGTGCTG | | | *OpHIS3* upstream  forward | | |
| P182 | CTTTATTGCTCAGCTGGGACAG | | | *OpHIS3* upstream  reverse | | |
| Primers for construction of pWYE3233 | | | | | | |
| P183 | ATTTTGGTCATGCATGAGATCAGATCTCGGCTTTCAGTTCTATATAC | | | *OpURA3* downstream  forward | | |
| P184 | GCATGCACTAGTGGTACCTGATTTGGAGAAATTGGAGAAG | | | *OpURA3* downstream  reverse | | |
| P185 | GGTACCACTAGTGCATGCAAGTGATATCTCAAGTTCCCAAG | | | *OpURA3* upstream  forward | | |
| P186 | CTTTTCAAAGA GTTGATTATTATTCAGGGAAATG | | | *OpURA3* upstream  reverse | | |
| P187 | AATAATCAACTCTTTGAAAAGATAATGTATG | | | *P_ScSNR52_* forward | | |
| P188 | GCTCTAAAACACGCGTGTGGGGTTTCAAGC GATCATTTATCTTTCACTGCG | | | *P_ScSNR52_* reverse | | |
| P189 | GATAAATGATC GCTTGAAACCCCACACGCGT GTTTTAGAGCTAGAAATAGC | | | crRNA forward | | |
| P190 | CATTTTGAAGCTATGGTGTGTGGGGGATCC AGACATAAAAAACAAAAAAA GCACC | | | crRNA reverse | | |
| Primers for deletion of *OpADE2* by CRISPR-Cas9 system | | | | | | |
| P191 | CTGAGGTTACGTAATATGCACTA | | | *OpADE2* 50-bp UHA  forward | | |
| P192 | TAATTTAACCTCTATGCTGACTTGGATATTATTATCTATG | | | *OpADE2* 50-bp UHA  reverse | | |
| P193 | AATATCCAAGTCAGCATAGAGGTTAAATTAATTCAATTAC | | | *OpADE2* 50-bp DHA  forward | | |
| P194 | TTTATTTTCAAAAAATAAATGC | | | *OpADE2* 50-bp DHA  reverse | | |
| P195 | CAAGTACTACTTCGAGGACGGCG | | | *OpADE2* 100-bp UHA  forward | | |
| P196 | TAATTTAACCTCTATGCTGACTTGGATATTATTATCTATG | | | *OpADE2* 100-bp UHA  reverse | | |
| P197 | AATATCCAAGTCAGCATAGAGGTTAAATTAATTCAATTAC | | | *OpADE2* 100-bp DHA  forward | | |
| P198 | GTGGAAAAATATCGAACGTGACTG | | | *OpADE2* 100-bp DHA  reverse | | |
| P199 | CTATACGTACTGTTCAGATACTTC | | | *OpADE2* 250-bp UHA  forward | | |
| P200 | TAATTTAACCTCTATGCTGACTTGGATATTATTATCTATG | | | *OpADE2* 250-bp UHA  reverse | | |
| P201 | AATATCCAAGTCAGCATAGAGGTTAAATTAATTCAATTAC | | | *OpADE2* 250-bp DHA  forward | | |
| P202 | GTCGTATCTCGTAAGTTGATTTAGG | | | *OpADE2* 250-bp DHA  reverse | | |
| P203 | GTCAGAAGTCAACAAGATCCAGG | | | *OpADE2* 500-bp UHA  forward | | |
| P204 | TAATTTAACCTCTATGCTGACTTGGATATTATTATCTATG | | | *OpADE2* 500-bp UHA  reverse | | |
| P205 | AATATCCAAGTCAGCATAGAGGTTAAATTAATTCAATTAC | | | *OpADE2* 500-bp DHA  forward | | |
| P206 | CCCGACCTCACCTTTACGACCTTC | | | *OpADE2* 500-bp DHA  reverse | | |
| P207 | GCCCTACTCCGGAACCATGGTCTC | | | *OpADE2* 750-bp UHA  forward | | |
| P208 | TAATTTAACCTCTATGCTGACTTGGATATTATTATCTATG | | | *OpADE2* 750-bp UHA  reverse | | |
| P209 | AATATCCAAGTCAGCATAGAGGTTAAATTAATTCAATTAC | | | *OpADE2* 750-bp DHA  forward | | |
| P210 | CATCATGGTTGTCCCAACAGGAAC | | | *OpADE2* 750-bp DHA  reverse | | |
| P211 | CGTACGAGGGCGACCAGCTCTCCG | | | *OpADE2* 1000-bp UHA  forward | | |
| P212 | TAATTTAACCTCTATGCTGACTTGGATATTATTATCTATG | | | *OpADE2* 1000-bp UHA  reverse | | |
| P213 | AATATCCAAGTCAGCATAGAGGTTAAATTAATTCAATTAC | | | *OpADE2* 1000-bp DHA  forward | | |
| P214 | GACACGTTCATTGAGGTCTACG | | | *OpADE2* 1000-bp DHA  reverse | | |
| Primers for detection of *OpADE2* deletion | | | | | | |
| P215 | CAAATCCTCACGCTTTCGCGC | | | *OpADE2*  forward | | |
| P216 | CCTTGGATGGGAAACACGTCATCC | | | *OpADE2* reverse | | |
| Primers for amplifications of potential off-target sites in OP040 (OP001 *OpURA3*^G73T^) | | | | | | |
| P217 | TCTGCGTTCTGGGCATAGAGACG | | | siteA forward | | |
| P218 | CCCCCACACCCAAAATTGTGCAG | | | siteA reverse | | |
| P219 | TCGTATATTTTCATCGTCTTTGCCG | | | siteB forward | | |
| P220 | TGCGCGGATAATGACGCCGGAGGAT | | | siteB reverse | | |
| P221 | TGGTACGTCCAATTCTTGCAGAG | | | siteC forward | | |
| P222 | GTTGGCGAGGATTTGAAGGATCTC | | | siteC reverse | | |
| P223 | TTGTCTGGTTATACTGATTCTGCGC | | | siteD forward | | |
| P224 | CCCGGCATGGCTGAAAACGCTCAGG | | | siteD reverse | | |
| P225 | TGCCGTCGTTAGGCCAGTACAAGC | | | siteE forward | | |
| P226 | CCAGCAGCAGTTCTGCAATGCTGAC | | | siteE reverse | | |
| P227 | CTCAGTCTGCGGCCGCGGCATACG | | | siteF forward | | |
| P228 | TTCCTTTAGTATGGAGGACACAAGC | | | siteF reverse | | |
| P229 | GGACATGGTCACCCGGCTAGAAGGG | | | siteG forward | | |
| P230 | TCGTCCATGAGTGGAATGATATACC | | | siteG reverse | | |

**Table S3** Potential off-target sites of CRISPR-Cas9 mediated point mutation in the gene *OpURA3.*

| Site | Bulge Type | Target | Chromosome | Position | Direction | Mismatches | Bulge Size |
| --- | --- | --- | --- | --- | --- | --- | --- |
| A | RNA | crRNA: CAGCAGACTACTTAATTTGANGG DNA: CAGCA-ACTtCaTAATTTGACGG | AECK01000007.1 | 258798 | - | 2 | 1 |
|  | RNA | crRNA: CAGCAGACTACTTAATTTGANGG   DNA: CAGCAa-CTtCaTAATTTGACGG | AECK01000007.1 | 258798 | - | 3 | 1 |
|  | RNA | crRNA: CAGCAGACTACTTAATTTGANGG   DNA: CAGC-aACTtCaTAATTTGACGG | AECK01000007.1 | 258798 | - | 3 | 1 |
| B | RNA | crRNA: CAGCAGACTACTTAATTTGANGG   DNA: C--CAGACTcCTTcATTTGATGG | AECK01000007.1 | 438370 | - | 2 | 2 |
|  | RNA | crRNA: CAGCAGACTACTTAATTTGANGG   DNA: Cc--AGACTcCTTcATTTGATGG | AECK01000007.1 | 438370 | - | 3 | 2 |
|  | X | crRNA: CAGCAGACTACTTAATTTGANGG   DNA: CAcCAGACTcCTTcATTTGATGG | AECK01000007.1 | 438370 | - | 3 | 0 |
| C | RNA | crRNA: CAGCAGACTACTTAATTTGANGG DNA: CAGCAGAaTACcTAATTT-AGGG | AECK01000004.1 | 649171 | - | 2 | 1 |
|  | RNA | crRNA: CAGCAGACTACTTAATTTGANGG   DNA: CAGCAGAaTACcTAA-TTtAGGG | AECK01000004.1 | 649171 | - | 3 | 1 |
|  | RNA | crRNA: CAGCAGACTACTTAATTTGANGG DNA: CAGCAGAaTACcTAAT-TtAGGG | AECK01000004.1 | 649171 | - | 3 | 1 |
|  | RNA | crRNA: CAGCAGACTACTTAATTTGANGG DNA: CAGCAGAaTACcTAATT-tAGGG | AECK01000004.1 | 649171 | - | 3 | 1 |
|  | RNA | crRNA: CAGCAGACTACTTAATTTGANGG   DNA: CAGCAGAaTACcTAATT--tAGG | AECK01000004.1 | 649172 | - | 3 | 2 |
| D | RNA | crRNA: CAGCAGACTACTTAATTTGANGG DNA: CAGCgGtCTACaTAATTT-ATGG | AECK01000005.1 | 226143 | + | 3 | 1 |
| E | RNA | crRNA: CAGCAGACTACTTAATTTGANGG DNA: CgGCAGACaAC--AATcTGACGG | AECK01000006.1 | 55550 | + | 3 | 2 |
| F | RNA | crRNA: CAGCAGACTACTTAATTTGANGG   DNA: CAGCAGAtT--TaAtTTTGATGG | AECK01000003.1 | 932728 | + | 3 | 2 |
| G | RNA | crRNA: CAGCAGACTACTTAATTTGANGG   DNA: CAGCAGA-TAgcTAtTTTGATGG | AECK01000004.1 | 75822 | + | 3 | 1 |

**Table S4** Editing efficiencies mediated by CRISPR-Cas9 in *O. polymorpha*

| gene locus | Type of gene modification | length of HA (bp) | correct/analyzed colonies | | | editing efficiency (%) |
| --- | --- | --- | --- | --- | --- | --- |
| *OpLEU2* | deletion | ~1000/1000 | 4/8 | 5/8 | 4/8 | 58.33 ± 7.22 |
|  | integration | ~1000/1000 | 5/8 | 5/8 | 5/8 | 62.50 |
| *OpURA3* | deletion | ~1000/1000 | 6/8 | 6/8 | 5/8 | 65.28 ± 2.41 |
|  | point mutation | ~1000/1000 | 14/52 | 18/52 | 17/52 | 31.40 ± 4.02 |
|  | integration | ~1000/1000 | 6/8 | 5/8 | 5/8 | 66.70 ± 7.22 |
| *OpHIS3* | integration | ~1000/1000 | 5/8 | 5/8 | 6/8 | 66.70 ± 7.22 |
| *OpLEU2& OpURA3& OpHIS3* | Multi-locus integration | ~1000/1000 | 7/24 | 7/24 | 8/24 | 30.56 ± 2.40 |
| *Op*rDNA | Multi-copy integration | ~1000/1000 | 18/24 | 21/24 | 15/24 | 75.00 ± 12.5 |
| *Sc*rDNA | Multi-copy integration | ~1000/1000 | 12/24 | 9/24 | 12/24 | 45.83 ± 7.22 |
| *OpADE2* | deletion | ~1000/1000 | 32/52 | 36/52 | 29/52 | 62.18 ± 6.17 |
|  | deletion | ~750/750 | 27/52 | 28/52 | 24/52 | 50.64 ± 4.00 |
|  | deletion | ~500/500 | 17/52 | 23/52 | 18/52 | 37.18 ± 6.18 |
|  | deletion | ~250/250 | 4/52 | 6/52 | 7/52 | 10.90 ± 2.94 |
|  | deletion | ~100/100 | 1/52 | 2/52 | 4/52 | 4.49 ± 2.94 |
|  | deletion | ~50/50 | 0/52 | 0/52 | 0/52 | 0 |
| *OpLEU2& OpURA3& OpHIS3* | Multi-locus deletion | ~1000/1000 | 4/24 | 6/24 | 7/24 | 23.61 ± 6.36 |

The ± error indicates standard deviations of three biological repeats, n=3.

**Table S5** Editing efficiencies mediated by endogenous HRS in *O. polymorpha.*

| gene locus | Type of gene modification | length of HA (bp) | correct/analyzed colonies | | | editing efficiency (%) |
| --- | --- | --- | --- | --- | --- | --- |
| *OpADE2* | deletion | ~1500/1500 | 14/52 | 14/52 | 18/52 | 29.49 ± 4.44% |
| *OpLEU2* | deletion | ~1000/1000 | 8/52 | 8/52 | 9/52 | 16.03 ± 1.11% |
| *OpHIS3* | deletion | ~1000/1000 | 9/52 | 9/52 | 8/52 | 16.67 ± 1.11% |
| *OpURA3* | deletion | ~1000/1000 | 7/52 | 9/52 | 12/52 | 17.95 ± 4.84% |

The ± error indicates standard deviations of three biological repeats, n=3.

**Table S6**. **Gene editing efficiencies of CRISPR-Cas9-assisted genome engineering methods in different yeasts**

| **Yeasts** | **Editing efficiency** | | | | | **Reference** |
| --- | --- | --- | --- | --- | --- | --- |
|  | Deletion | Point  mutation | Single site integration | Multi-locus integration | Multi-copy integration |  |
| *O. polymorpha* | 58.33±7.22%  (*OpLEU2*)  65.28±2.41% (*OpURA3*)  Multi-locus integration  23.61 ± 6.36%  (*OpLEU2& OpURA3& OpHIS3* | 31.40±4.02%  (*OpURA3*) | 66.70% (*OpHIS3*::*gfpmut3a,*  66.70±7.22%  *OpURA3*::*gfpmut3a*)  62.50% (*OpLEU2*::*gfpmut3a*) | 30.56±2.40%  (*OpURA3*::*TAL,OpHIS3*::*4CL,*  *OpLEU2*::*STS*) | 75.00±12.5%  (*Op*rDNA)  45.83±7.22%  (*Sc*rDNA) | This study |
|  | 9% (disruption of *OpADE2*) | **—** | **—** | **—** | **—** | Juergens *et al.* , 2018^2^ |
|  | 47% (*OpADE12*)  50%, 71%  (disruptions of *OpPHO1*)  17%, 30%  (disruptions of  *OpPHO11*) | **—** | **—** | **—** | **—** | Numamoto *et al.* , 2017[[1](#_ENREF_1)] |
| *S. cerevisiae* | **—** | ~100% (*OpADE2*) | 99.03%  (*CAN1*::*KanMX*) | **—** | **—** | DiCarlo *et al.*, 2013[[15](#_ENREF_15)] |
|  | **—** | **—** | 96%  (*OpADE2*::*crtB*), 77%  (*OpURA3*::*crtE*), 79.2%  (*OpHIS3*::*crtI*) | 30.6%  (*OpURA3*::*crtE OpHIS3*::*crtI*  *OpADE2*::*crtB*) | **—** | Bao *et al.，*2015[[14](#_ENREF_14)] |
|  | **—** | **—** | 100% (*X-2*::*tHMG1*) | 84%  (*X-3*::*crtI*  *XI-2*::*crtYB*  *XII-5*::*BTS1*) | **—** | Ronda *et al.*, 2015[[16](#_ENREF_16)] |
|  | 91%  (*RHR2*, *ADH5*, *HO*) | 90-100%  (11  genes) | **—** | 4.2% (*GAL80*, *HO*,  *ARO1*) | **—** | Horwitz *et al.*, 2015[[17](#_ENREF_17)] |
|  | **—** | **—** | **—** | **—** | ~80%  (*Ty* elements) | Shi *et al.*, 2016[[18](#_ENREF_18)] |
| *Pichia pastoris* | 78.3% (*GUT1*) | **—** | **—** | **—** | **—** | Weninger *et al.*, 2016[[19](#_ENREF_19)] |
| *Yarrowia lipolytica* | 64%  (*MFE1*) | **—** | **—** | **—** | **—** | Schwartz *et al.*, 2016[[20](#_ENREF_20)] |
|  | 62.5% (*PEX10*) | **—** | **—** | **—** | **—** | Gao *et al.*, 2016[[21](#_ENREF_21)] |
|  | **—** | **—** | 0-69% (17 sites) | **—** | **—** | Schwartz *et al.* , 2017[[22](#_ENREF_22)] |
| *Schizosaccharomyces Pombe* | 85-98% (*ADE6*) | **—** | **—** | **—** | **—** | Jacobs *et al.*, 2014[[23](#_ENREF_23)] |
| *Candida albicans* | ~80% (*OpLEU2*) | **—** | **—** | **—** | **—** | Ng *et al.*, 2017[[24](#_ENREF_24)] |
| *Cryptococcus neoformans* | 87.64% (*OpADE2*) | **—** | **—** | **—** | **—** | Wang *et al.* , 2016[[25](#_ENREF_25)] |


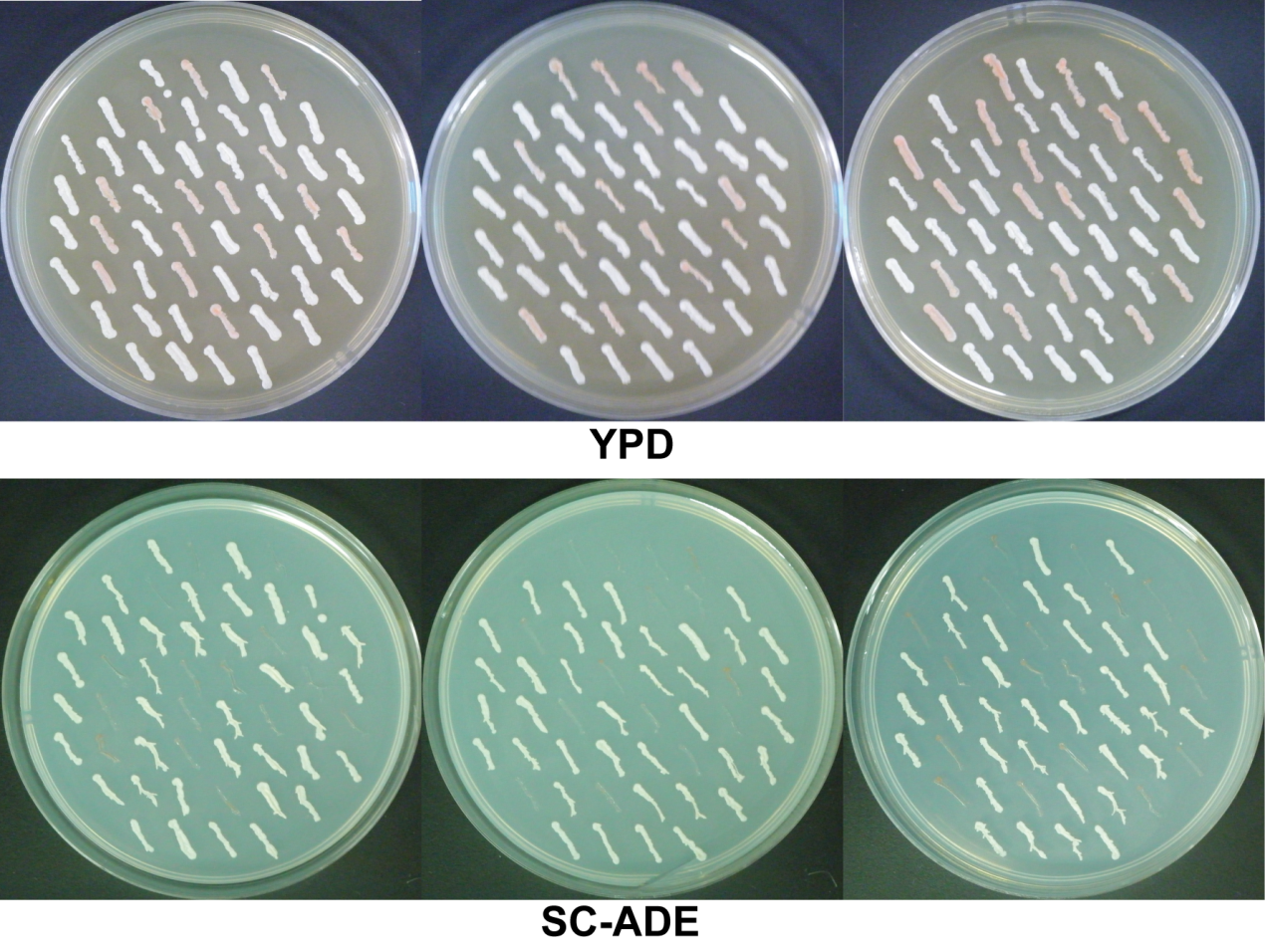


Figure S1 Analysis of editing efficiency mediated by endogenous homologous recombination system at the gene *OpADE2* site by cell growth phenotype on YPD and SC without adenine (SC-ADE) plates. Wild type stains grew normally on the SC-ADE plates, while mutants failed to grow without adenine.


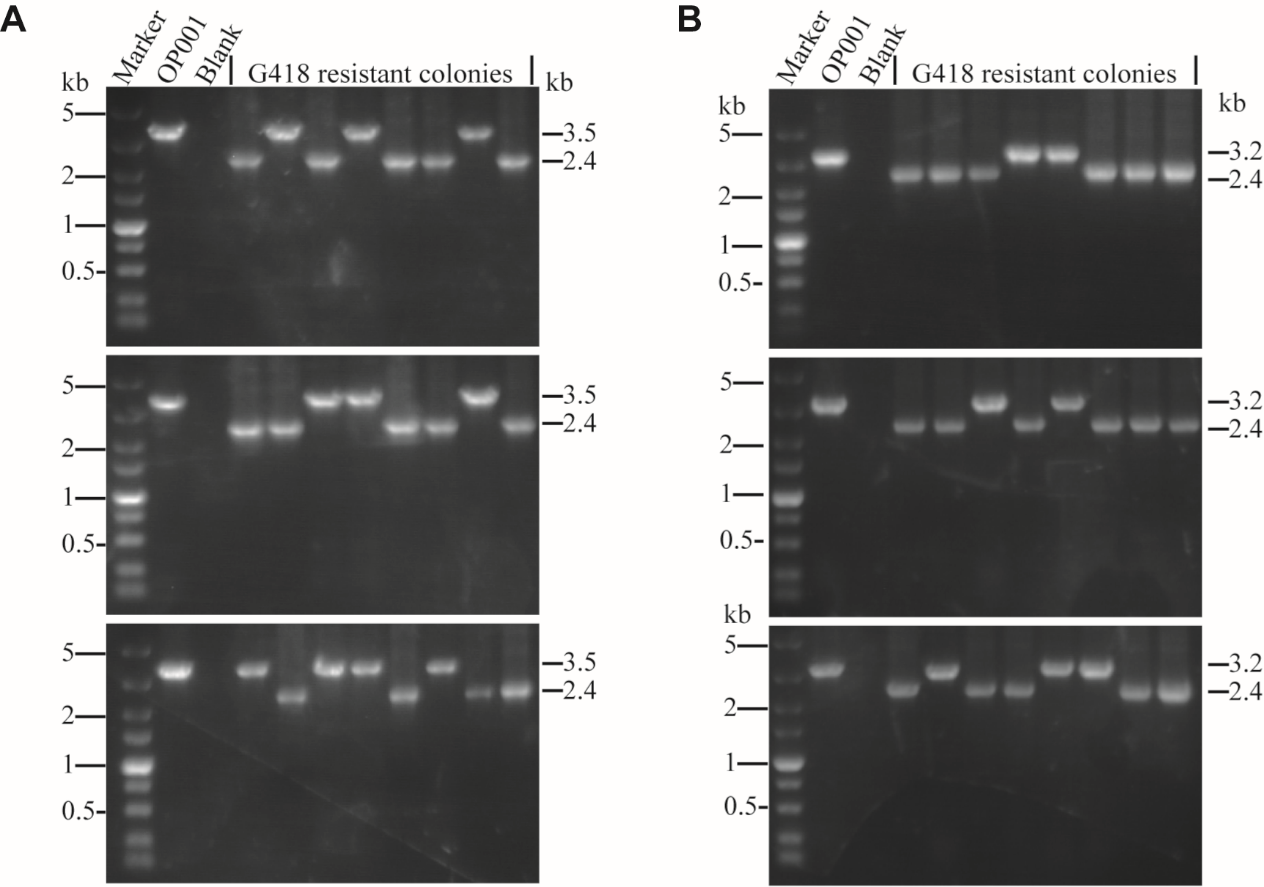


Figure S2 PCR identifications for the deletion of *OpLEU2* (A) and *OpURA3* (B) genes, respectively. A blank without the template was used as the control. The Δ*OpLEU2* mutant yielded a 2386-bp fragment, while the OP001 yielded a 3475-bp fragment. The Δ*OpURA3* mutant yielded a 2444-bp fragment, while the OP001 yielded a 3236-bp fragment.


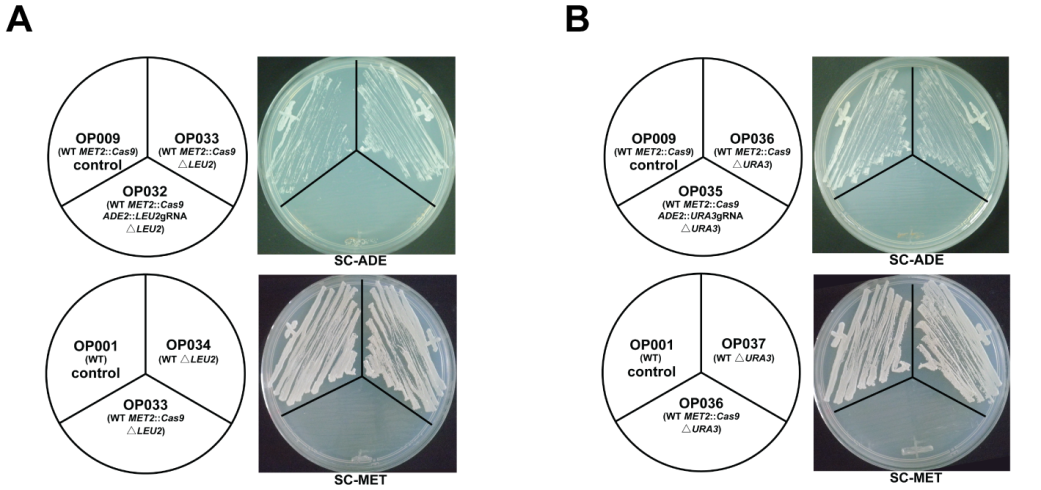


Figure S3 Evictions of the linearized gRNA delivery vector and the linearized Cas9 protein expression vector after gene editing. (A) Evictions of pWYE3209 and pWYE3208. After the *OpLEU2* gene was deleted, successive evictions of the linearized *OpLEU2*-gRNA delivery vector pWYE3209 and the linearized Cas9 expression vector pWYE3208 were identified by cell growth phenotype on SC-ADE plates and SC-MET plates, respectively. (B) Evictions of pWYE3211 and pWYE3208. After the *OpURA3* gene was deleted, the successive evictions of the linearized *OpURA3*-gRNA^*^ delivery vector pWYE3211 and the linearized Cas9 expression vector pWYE3208 were identified by cell growth phenotype on SC-ADE plates and SC-MET plates respectively.


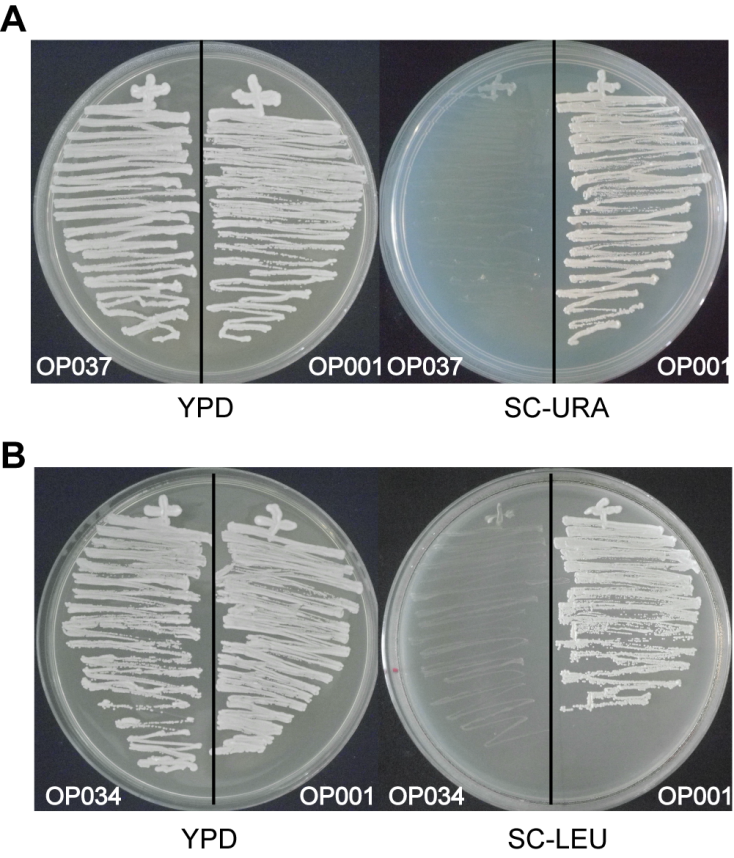


**Figure S4** Verification of gene deletions by auxotrophic phenotype analysis. (A) Verification of *OpURA3* deletion using SC-URA plates assay. (B) Verification of *OpLEU2* deletion using SC-LEU plates assay.


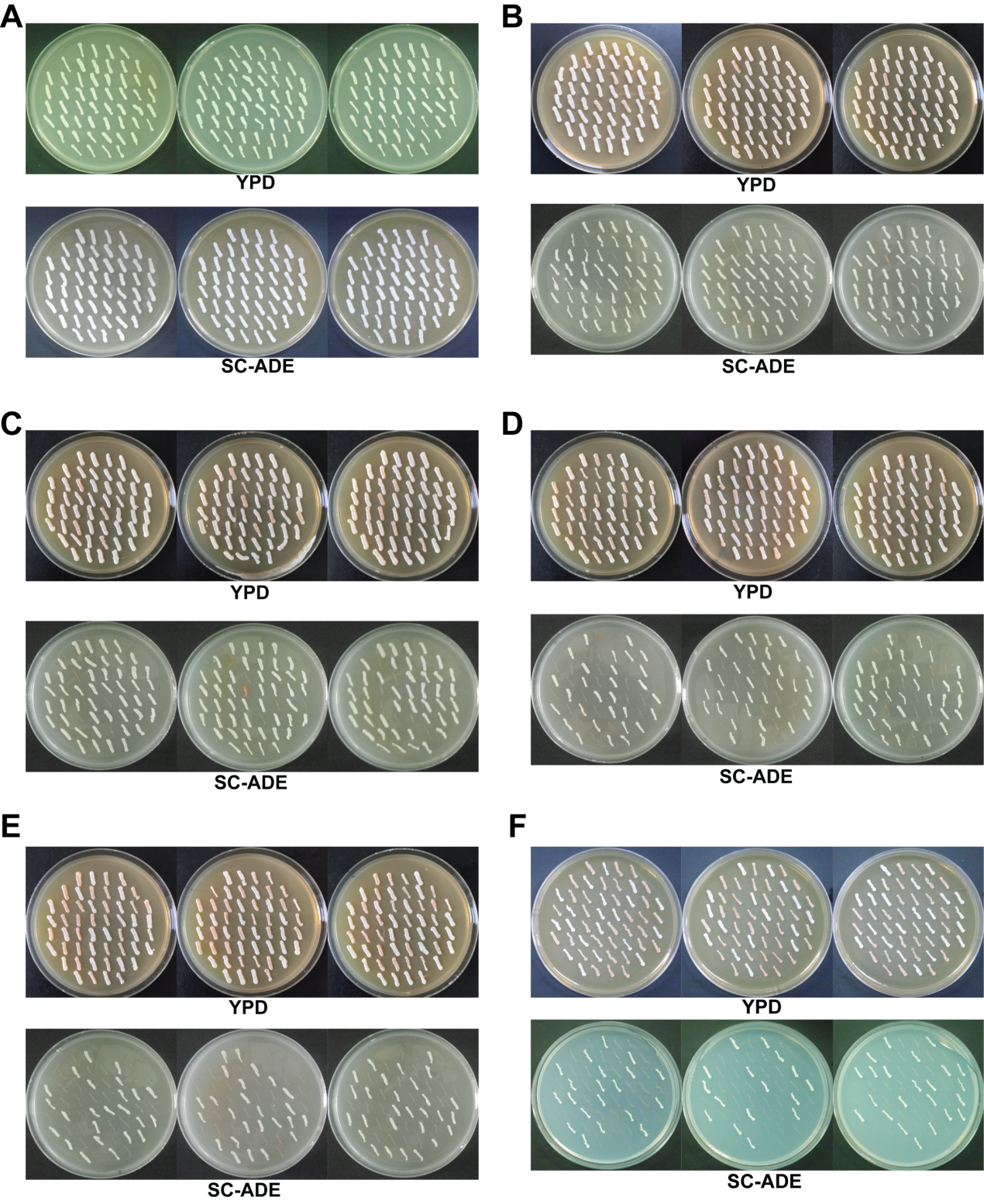


Figure S5 Effect of HA (homologous arm) on editing efficiency of CRISPR-Cas9 mediated gene deletion in *O. polymorpha*. Editing templates containing HAs of 50-bp (A), 100-bp (B), 250-bp (C), 500-bp (D), 750-bp (E), and 1,000-bp (F) were used respectively. Editing efficiency was evaluated by phenotypical screening. Wild type stains grew normally on the SC-ADE plates, while mutants failed to grow without adenine.


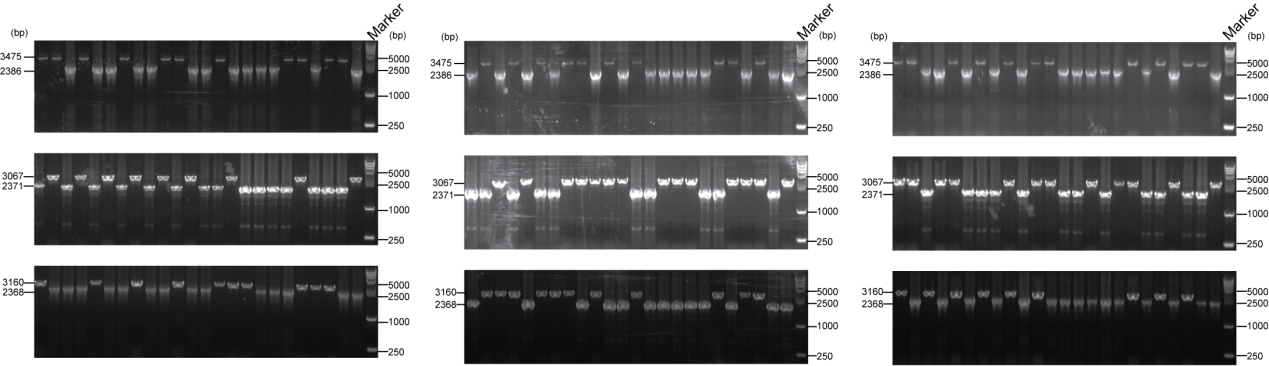


**Figure S6** PCR identifications of multi-locus simultaneous knock-outs of genes *OpLEU2*, *OpHIS3* and *OpURA3*. The Δ*OpLEU2* mutant yielded a 2386-bp fragment, while the OP001 yielded a 3475-bp fragment. The Δ*OpHIS3* mutant yielded a 2371-bp fragment, while the OP001 yielded a 3067-bp fragment. The Δ*OpURA3* mutant yielded a 2368-bp fragment, while the OP001 yielded a 3160-bp fragment.


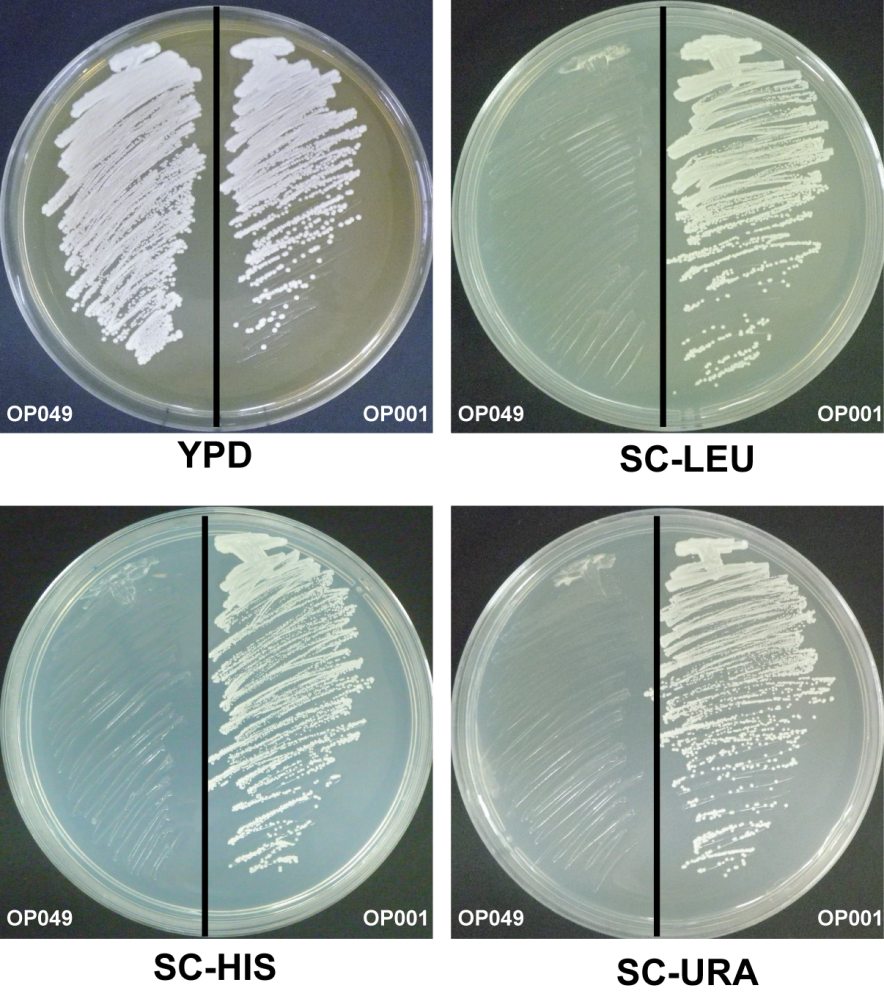


**Figure S7** Verification of multi-locus simultaneous knock-outs by auxotrophic phenotype analysis. Wild type stains grew normally on the SC-LEU, SC-HIS and SC-URA plates, while the mutant ()failed to grow on these plates.


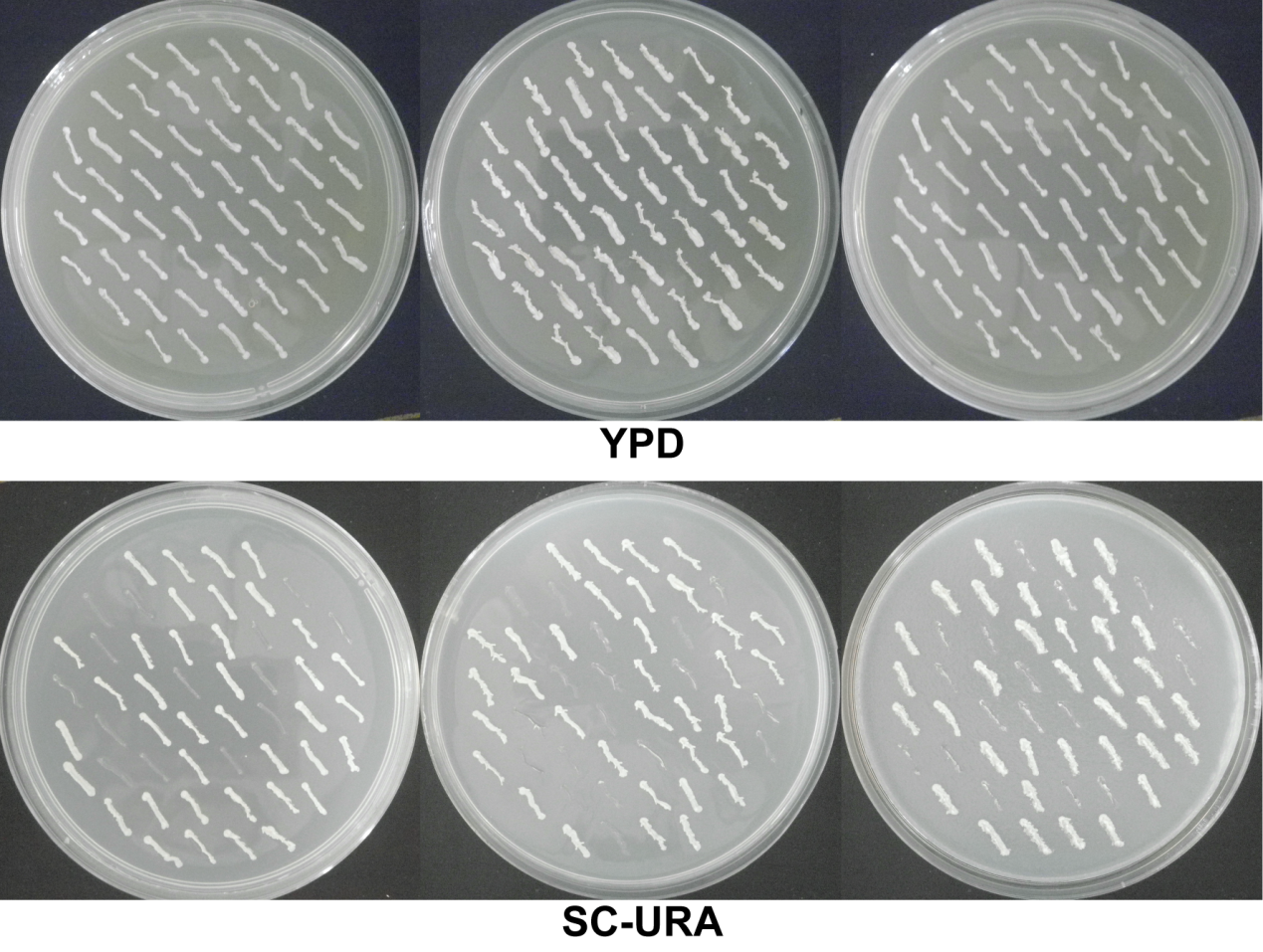


Figure S8 The identification of the point mutation by cell growth phenotype on YPD and SC without uracil (SC-URA) plates. Wild type stains grew normally on the SC-URA plates, while mutants failed to grow without uracil.


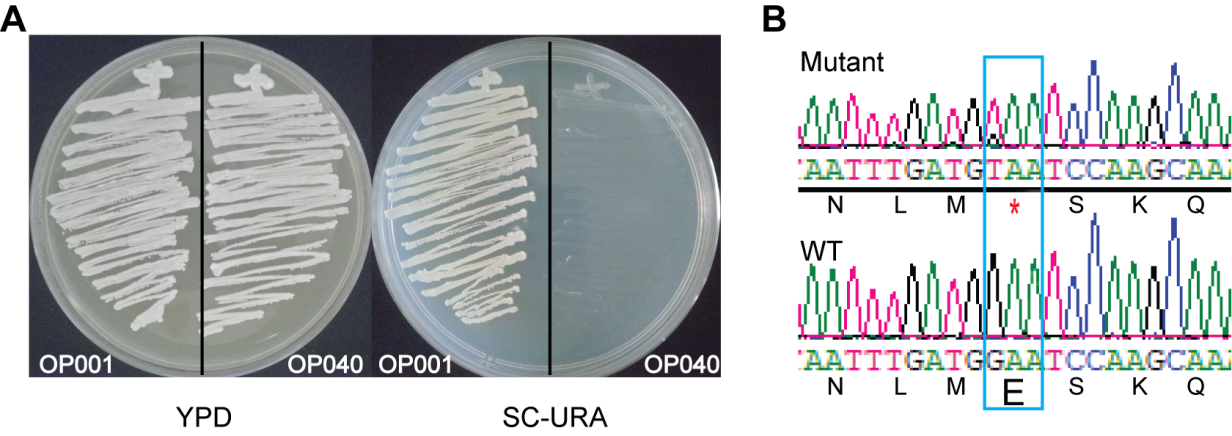


Figure S9 Verifications of point mutation of the gene *OpURA3* in the mutant OP040 (OP001 *OpURA3*^G73T^) by auxotrophic phenotype analysis (A) and DNA sequencing (B).


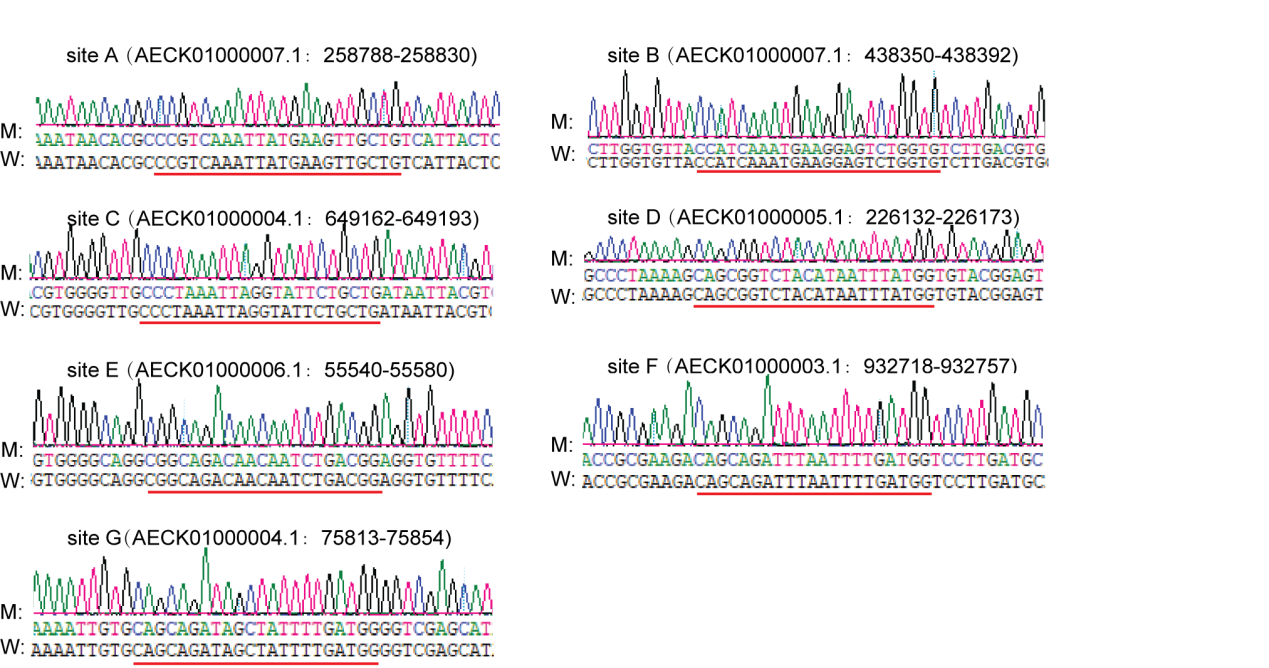


Figure S10 DNA sequencing of similar genomic loci of point mutation site in the gene *OpURA3 of* the mutant OP040 (OP001 *OpURA3*^G73T^)*.* M: nucleotide sequence in the mutant OP040. W: nucleotide sequence in wildtype OP001. The precise genomic positions of every site were included in parentheses. The sequences similar to *OpURA3*gRNA* were underlined.


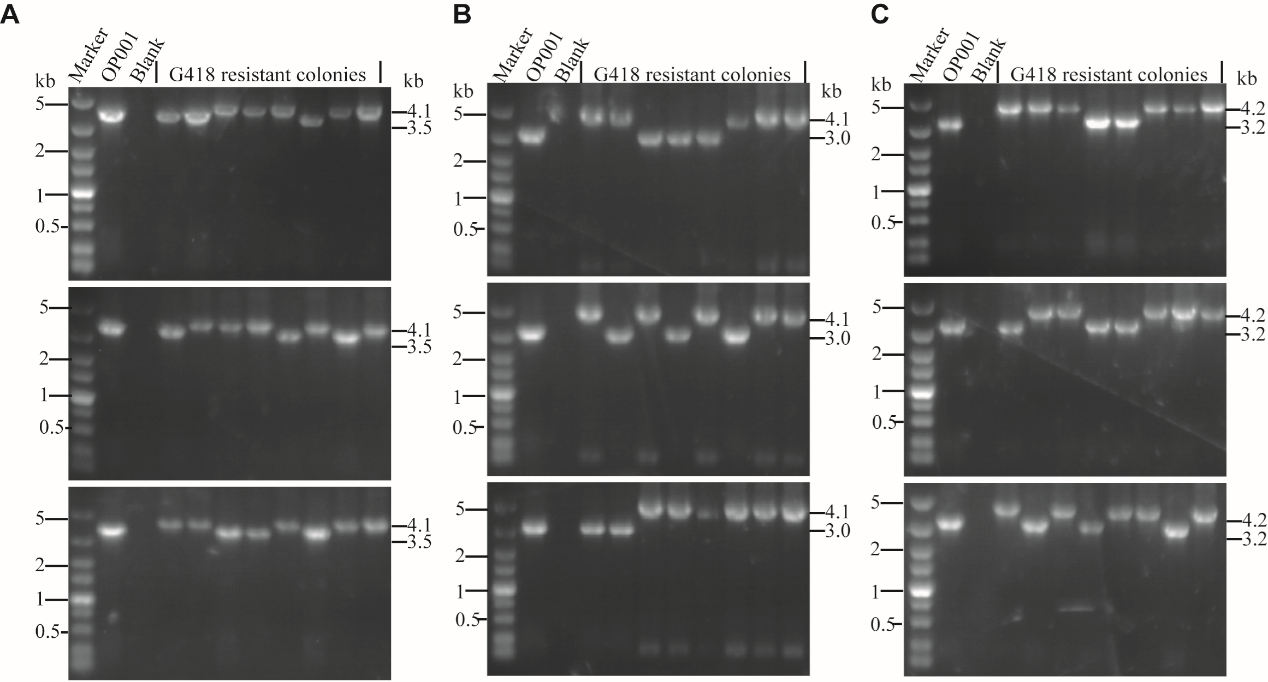


Figure S11 PCR identifications of *gfpmut3a* expression cassette integration at *OpLEU2* (A), *OpHIS3* (B) and *OpURA3* (C) loci. A blank without the template was used as the control. The Δ*OpLEU2*::*gfpmut3a* mutant yielded a 4140-bp fragment, while the OP001 yielded a 3475-bp fragment. The Δ*OpHIS3*::*gfpmut3a* mutant yielded a 4101-bp fragment, while the OP001 yielded a 3043-bp fragment. The Δ*OpURA3*::*gfpmut3a* mutant yielded a 4198-bp fragment, while the OP001 yielded a 3236-bp fragment.


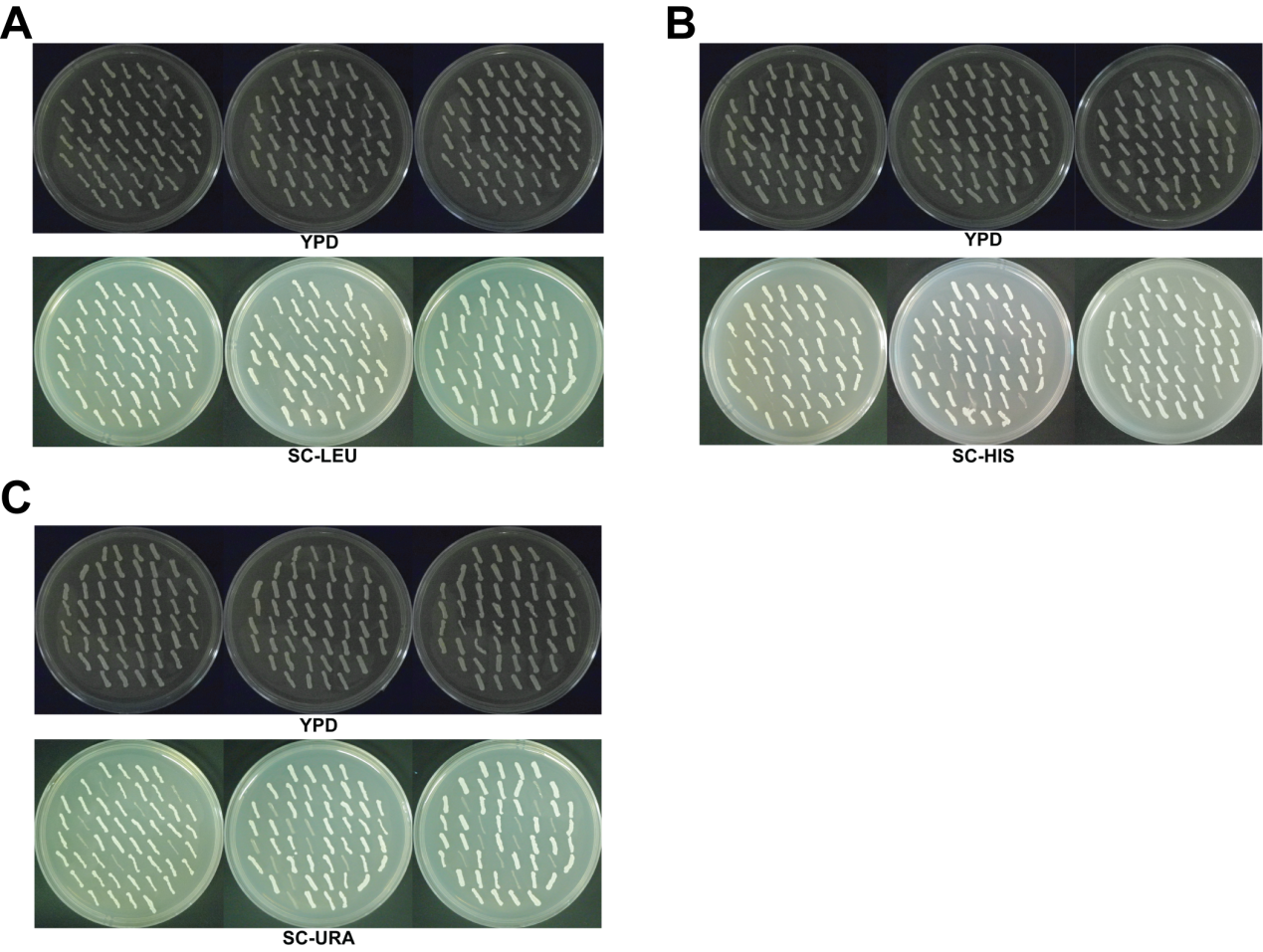


Figure S12 Analysis of editing efficiencies mediated by endogenous homologous recombination system at the gene *OpLEU2* (A), *OpHIS3* (B) and *OpURA3* (C) sites by cell growth phenotype. Wild type stains grew normally on the SC-LEU, SC-HIS and SC-URA plates. While the Δ*OpLEU2* mutants failed to grow without leucine (A). Δ*OpHIS3* mutants failed to grow without histidine (B). Δ*OpURA3* mutants failed to grow without uracil (C)


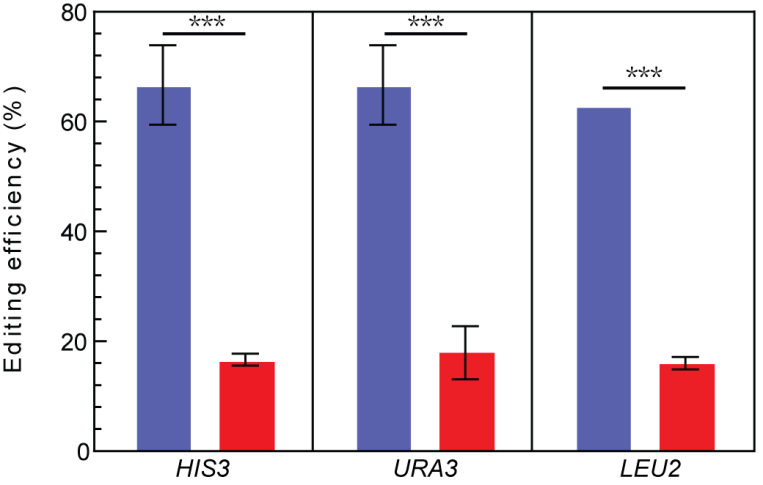


Figure S13 A comparison of  the editing efficiencies at three gene sites by two different methods. The blue bars represent the editing efficiencies of CRISPR-Cas9-assisted genome editing method, while the red bars represent that of PCR fragments mediated genome editings. ****P* < 0.001, determined by two-tailed Student t-test. Error bars represent standard deviations.


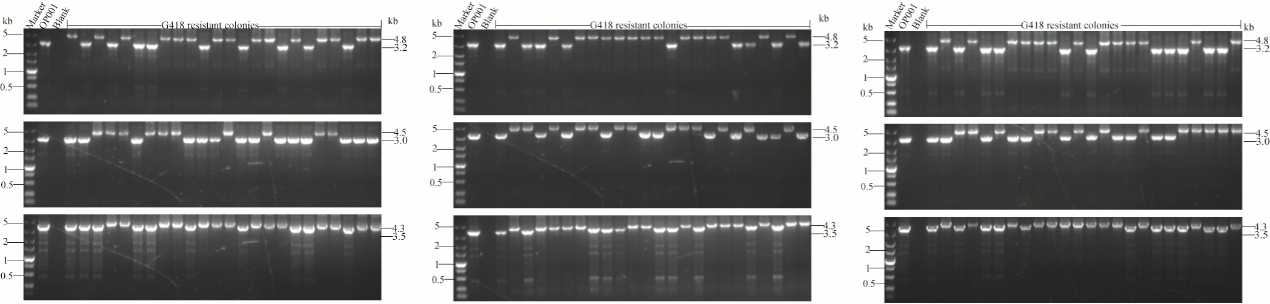


Figure S14 PCR identifications of simultaneously multi-loci genomic integration. A blank without the template was used as the control. The Δ*OpURA3*::*TAL* mutant yielded a 4762-bp fragment, while the OP001 yielded a 3236-bp fragment. The Δ*OpHIS3*::*4CL* mutant yielded a 4522-bp fragment, while the OP001 yielded a 3043-bp fragment. The Δ*OpLEU2*::*STS* mutant yielded a 4275-bp fragment, while the OP001 yielded a 3475-bp fragment.

**
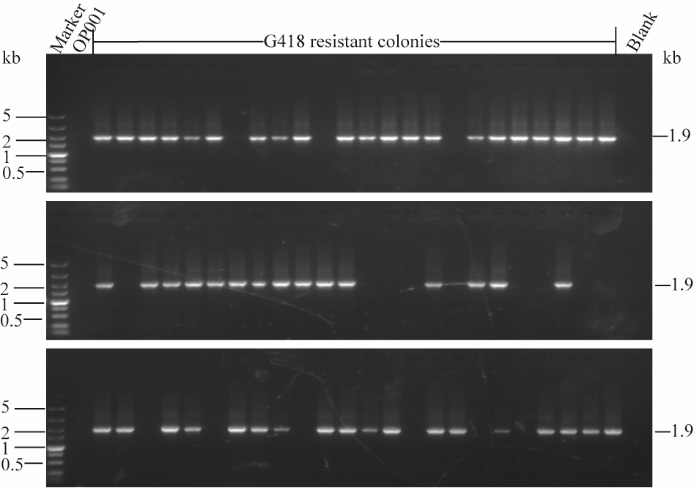
**

Figure S15 PCR identification of *gfpmut3a* expression cassette by multi-copy integration at rDNA cluster in *O. polymorpha*. A blank without the template and wild-type strain OP001were used as the control. An 1891-bp fragment was amplified from the integration mutant, while wild-type strains couldn’t yield fragment.


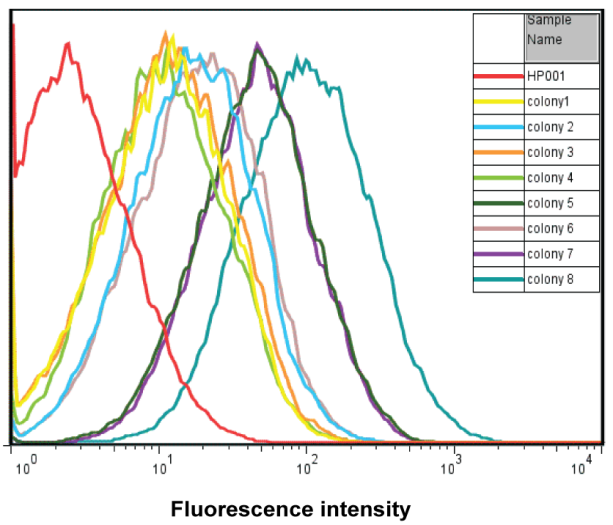


Figure S16 Flow cytometry analysis of the expression of GFP in eight randomly selected *O. polymorpha* colonies.

**
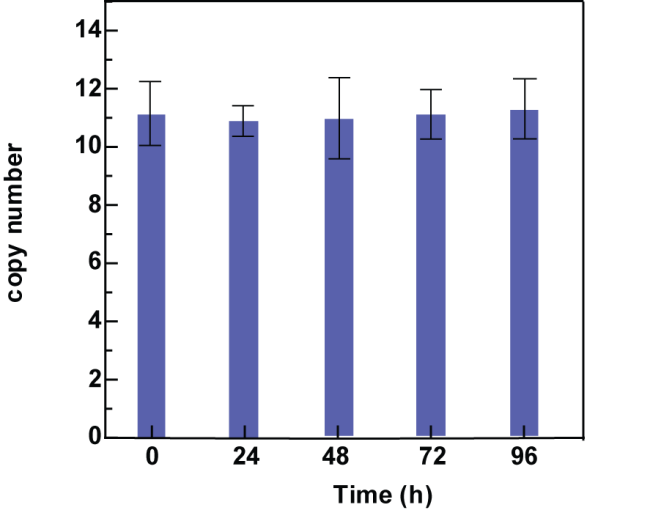
**

Figure S17 Stability of multi-copy integration of *gfpmut3a* at rDNA repeats in the mutant OP025 upon continuous culture for 96 hours.


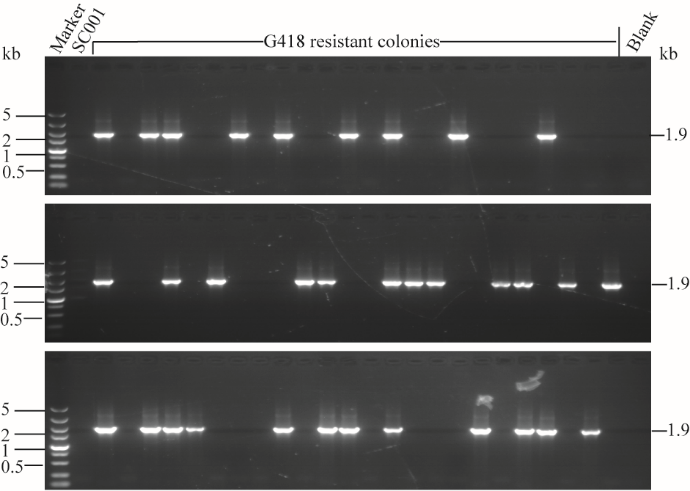


Figure S18 PCR identification of *gfpmut3a* expression cassette by multi-copy integration at rDNA cluster in *S. cerevisiae*. A blank without the template was used as the control. The integration mutant yielded a 1938-bp fragment, while the SC001 couldn’t yield fragment.


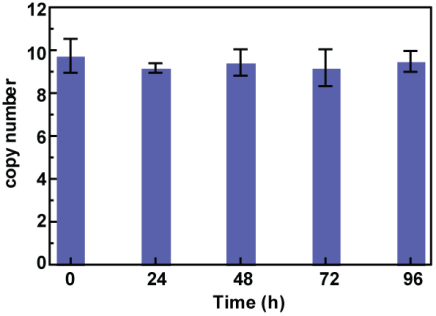


Figure S19 Stability of multi-copy integration of *gfpmut3a* at rDNA repeats in the mutant SC007 upon continuous culture for 96h.

**References**

1. Numamoto M, Maekawa H, Kaneko Y: Efficient genome editing by CRISPR/Cas9 with a tRNA-sgRNA fusion in the methylotrophic yeast *Ogataea polymorpha*. *J Biosci Bioeng* 2017.

2. Juergens H, Varela JA, Gorter de Vries AR, Perli T, Gast VJM, Gyurchev NY, Rajkumar AS, Mans R, Pronk JT, Morrissey JP *et al*: Genome editing in Kluyveromyces and Ogataea yeasts using a broad-host-range Cas9/gRNA co-expression plasmid. *FEMS yeast research* 2018, 18(3).

3. Saraya R, Krikken AM, Kiel JA, Baerends RJ, Veenhuis M, van der Klei IJ: Novel genetic tools for *Hansenula polymorpha*. *FEMS yeast research* 2012, 12(3):271-278.

4. Wagner JM, Alper HS: Synthetic biology and molecular genetics in non-conventional yeasts: Current tools and future advances. *Fungal Genet Biol* 2016, 89:126-136.

5. Bogdanova AI, Agaphonov MO, Ter-Avanesyan MD: Plasmid reorganization during integrative transformation in Hansenula polymorpha. *Yeast* 1995, 11(4):343-353.

6. Sohn JH, Choi ES, Kang HA, Rhee JS, Rhee SK: A family of telomere-associated autonomously replicating sequences and their functions in targeted recombination in *Hansenula polymorpha* DL-1. *J Bacteriol* 1999, 181(3):1005-1013.

7. Janowicz ZA, Melber K, Merckelbach A, Jacobs E, Harford N, Comberbach M, Hollenberg CP: Simultaneous expression of the S and L surface antigens of hepatitis B, and formation of mixed particles in the methylotrophic yeast, Hansenula polymorpha. *Yeast* 1991, 7(5):431-443.

8. Gatzke R, Weydemann U, Janowicz ZA, Hollenberg CP: Stable multicopy integration of vector sequences in *Hansenula polymorpha*. *Appl Microbiol Biotechnol* 1995, 43(5):844-849.

9. Bharathi V, Girdhar A, Prasad A, Verma M, Taneja V, Patel BK: Use of ade1 and ade2 mutations for development of a versatile red/white colour assay of amyloid-induced oxidative stress in saccharomyces cerevisiae. *Yeast* 2016, 33(12):607-620.

10. Cheon SA, Choo J, Ubiyvovk VM, Park JN, Kim MW, Oh DB, Kwon O, Sibirny AA, Kim JY, Kang HA: New selectable host-marker systems for multiple genetic manipulations based on *TRP1*, *MET2* and *ADE2* in the methylotrophic yeast *Hansenula polymorpha*. *Yeast* 2009, 26(9):507-521.

11. Zhang G, Wang W, Deng A, Sun Z, Zhang Y, Liang Y, Che Y, Wen T: A mimicking-of-DNA-methylation-patterns pipeline for overcoming the restriction barrier of bacteria. *PLoS Genet* 2012, 8(9):e1002987.

12. Zacchi LF, Gomez-Raja J, Davis DA: Mds3 regulates morphogenesis in Candida albicans through the TOR pathway. *Mol Cell Biol* 2010, 30(14):3695-3710.

13. Song P, Liu S, Guo X, Bai X, He X, Zhang B: Scarless gene deletion in methylotrophic *Hansenula polymorpha* by using *mazF* as counter-selectable marker. *Anal Biochem* 2014, 468C:66-74.

14. Bao Z, Xiao H, Liang J, Zhang L, Xiong X, Sun N, Si T, Zhao H: Homology-integrated CRISPR-Cas (HI-CRISPR) system for one-step multigene disruption in *Saccharomyces cerevisiae*. *ACS synthetic biology* 2015, 4(5):585-594.

15. DiCarlo JE, Norville JE, Mali P, Rios X, Aach J, Church GM: Genome engineering in *Saccharomyces cerevisiae* using CRISPR-Cas systems. *Nucleic Acids Res* 2013, 41(7):4336-4343.

16. Ronda C, Maury J, Jakociunas T, Jacobsen SA, Germann SM, Harrison SJ, Borodina I, Keasling JD, Jensen MK, Nielsen AT: CrEdit: CRISPR mediated multi-loci gene integration in *Saccharomyces cerevisiae*. *Microbial cell factories* 2015, 14:97.

17. Horwitz AA, Walter JM, Schubert MG, Kung SH, Hawkins K, Platt DM, Hernday AD, Mahatdejkul-Meadows T, Szeto W, Chandran SS *et al*: Efficient Multiplexed Integration of Synergistic Alleles and Metabolic Pathways in Yeasts via CRISPR-Cas. *Cell systems* 2015, 1(1):88-96.

18. Shi S, Liang Y, Zhang MM, Ang EL, Zhao H: A highly efficient single-step, markerless strategy for multi-copy chromosomal integration of large biochemical pathways in *Saccharomyces cerevisiae*. *Metabolic engineering* 2016, 33:19-27.

19. Weninger A, Hatzl AM, Schmid C, Vogl T, Glieder A: Combinatorial optimization of CRISPR/Cas9 expression enables precision genome engineering in the methylotrophic yeast Pichia pastoris. *J Biotechnol* 2016, 235:139-149.

20. Schwartz CM, Hussain MS, Blenner M, Wheeldon I: Synthetic RNA Polymerase III Promoters Facilitate High-Efficiency CRISPR-Cas9-Mediated Genome Editing in Yarrowia lipolytica. *ACS synthetic biology* 2016, 5(4):356-359.

21. Gao S, Tong Y, Wen Z, Zhu L, Ge M, Chen D, Jiang Y, Yang S: Multiplex gene editing of the Yarrowia lipolytica genome using the CRISPR-Cas9 system. *J Ind Microbiol Biotechnol* 2016, 43(8):1085-1093.

22. Schwartz C, Shabbir-Hussain M, Frogue K, Blenner M, Wheeldon I: Standardized Markerless Gene Integration for Pathway Engineering in *Yarrowia lipolytica*. *ACS synthetic biology* 2017, 6(3):402-409.

23. Jacobs JZ, Ciccaglione KM, Tournier V, Zaratiegui M: Implementation of the CRISPR-Cas9 system in fission yeast. *Nature communications* 2014, 5:5344.

24. Ng H, Dean N: Dramatic Improvement of CRISPR/Cas9 Editing in Candida albicans by Increased Single Guide RNA Expression. *mSphere* 2017, 2(2).

25. Wang Y, Wei D, Zhu X, Pan J, Zhang P, Huo L, Zhu X: A 'suicide' CRISPR-Cas9 system to promote gene deletion and restoration by electroporation in Cryptococcus neoformans. *Sci Rep* 2016, 6:31145.
